# Regulatory role of *Prunus mume* DAM6 on lipid body accumulation and phytohormone metabolism in the dormant vegetative meristem

**DOI:** 10.1093/hr/uhae102

**Published:** 2024-04-09

**Authors:** Tzu-Fan Hsiang, Hisayo Yamane, Mei Gao-Takai, Ryutaro Tao

**Affiliations:** Graduate School of Agriculture, Kyoto University, Kyoto 606-8502, Japan; Graduate School of Agriculture, Kyoto University, Kyoto 606-8502, Japan; Experimental Farm, Ishikawa Prefectural University, Nonoichi 921-8836, Japan; Graduate School of Agriculture, Kyoto University, Kyoto 606-8502, Japan

## Abstract

Bud dormancy is a crucial process in the annual growth cycle of woody perennials. In Rosaceae fruit tree species, *DORMANCY-ASSOCIATED MADS-box* (*DAM*) transcription factor genes regulating bud dormancy have been identified, but their molecular roles in meristematic tissues have not been thoroughly characterized. In this study, molecular and physiological analyses of transgenic apple plants overexpressing the Japanese apricot *DAM6* gene (*PmDAM6*) and Japanese apricot cultivars and F_1_ individuals with contrasting dormancy characteristics revealed the metabolic pathways controlled by PmDAM6. Our transcriptome analysis and transmission electron microscopy examination demonstrated that PmDAM6 promotes the accumulation of lipid bodies and inhibits cell division in the dormant vegetative meristem by down-regulating the expression of lipid catabolism genes (*GDSL ESTERASE/LIPASE* and *OIL BODY LIPASE*) and *CYCLIN* genes, respectively. Our findings also indicate PmDAM6 promotes abscisic acid (ABA) accumulation and decreases cytokinin (CTK) accumulation in vegetative buds by up-regulating the expression of the ABA biosynthesis gene *ARABIDOPSIS ALDEHYDE OXIDASE* and the CTK catabolism gene *CYTOKININ DEHYDROGENASE*, while also down-regulating the expression of the CTK biosynthesis genes *ISOPENTENYL TRANSFERASE* (*IPT*) and *CYP735A*. Additionally, PmDAM6 modulates gibberellin (GA) metabolism by up-regulating *GA2-OXIDASE* expression and down-regulating *GA3-OXIDASE* expression. Furthermore, PmDAM6 may also indirectly promote lipid accumulation and restrict cell division by limiting the accumulation of CTK and GA in buds. In conclusion, using our valuable genetic platform, we clarified how PmDAM6 modifies diverse cellular processes, including lipid catabolism, phytohormone (ABA, CTK, and GA) biosynthesis and catabolism, and cell division, in the dormant vegetative meristem.

## Introduction

Temperate and boreal perennials have evolved an annual growth cycle that includes periods of dormancy that enable them to survive in cold environments [1, 2]. After shoot growth ceases and terminal buds are set or aborted (self-pruning), endodormancy (i.e., period of arrested growth) begins. At the same time, the lateral buds transition from para-dormancy, during which lateral bud growth is suppressed by active apical shoot growth (i.e., apical dominance), to endodormancy [3]. Buds that have entered endodormancy are unable to resume growth until their chilling requirement has been fulfilled [4]. Furthermore, after the genotype-specific chilling requirement is satisfied, buds enter ecodormancy (also known as quiescence). Unlike endodormancy, ecodormancy involves growth inhibition caused by external environmental factors, including low temperatures and drought stress [5]. Hereafter, “dormancy” in this context refers to endodormancy.

 The molecular mechanisms regulating the bud dormancy of fruit trees in the family Rosaceae have been extensively studied, and *DORMANCY-ASSOCIATED MADS-box* (*DAM*) transcription factor genes were identified as important dormancy regulators [6–9]. The *DAM* genes were first identified as the causal genes in the peach (*Prunus persica*) *evergrowing* (*evg*), which fails to enter dormancy under dormancy-inducing environmental conditions and exhibits an annual evergrowing phenotype [10], suggesting *DAM* genes are involved in bud dormancy regulation [11, 12]. However, the molecular roles of Rosaceae DAMs have not been fully characterized by genetic studies. In a previous study on the RNAi-mediated repression of *MdDAM* and *MdSVP* expression that leads to the evergrowing phenotype in apple (*Malus* × *domestica*), the key abscisic acid (ABA) biosynthesis gene *9-CIS-EPOXYCAROTENOID DIOXYGENASE3* (*NCED3*) and *CALLOSE SYNTHASE* (*CALS*) were expressed at lower levels in the RNAi lines than in the wild-type (WT) control [13]. Although other reports suggested that Rosaceae DAMs regulate dormancy by directly modulating phytohormone metabolism [14], including ABA biosynthesis [15] and ABA signaling [16], the DAM target genes have only been characterized using non-meristematic tissues. Thus, there is an essential need for (1) determining whether the proposed DAM targets are actually affected by DAM in the bud vegetative meristem and (2) identifying additional DAM target genes in the vegetative bud meristem, with roles in the complex network regulating dormancy. These objectives may be achieved at least partly via the endogenous modulation of *DAM* expression in the vegetative bud meristem.

We previously observed that PmDAM6, which is encoded by one of six *DAM* genes in the *Prunus mume* genome, can inhibit growth and promote bud set in transgenic poplar (*Populus* spp.) plants [17]. We also determined that the overexpression of *PmDAM6* in transgenic apple (*Malus × domestica*) plants inhibits growth, adversely affects the bud break competency of dormant buds, and delays bud dormancy release. In another study, ABA and cytokinin (CTK) levels were significantly higher and lower, respectively, in *PmDAM6*-overexpressing lines than in the WT control [18]. These *DAM*-overexpressing lines may be useful for further clarifying the vegetative bud dormancy mechanism in Rosaceae. In addition to phytohormones, lipid bodies, carbon resources, reactive oxygen species, and certain metabolites may also be associated with dormancy. Therefore, further analyses of the physiological characteristics of *DAM*-overexpressing vegetative buds may provide valuable insights into the important metabolic pathways underlying Rosaceae vegetative bud dormancy.

The objective of this study was to identify the genes and metabolic pathways affected by PmDAM6 using vegetative buds from adult transgenic apple trees that we previously established [18]. These included *PmDAM6*-overexpressing (*35S:PmDAM6*) apple lines and dexamethasone (DEX)-inducible *PmDAM6*-overexpressing (*35S:PmDAM6-GR*) apple lines. The vegetative bud transcriptomes were comprehensively analyzed to identify potential target genes. Furthermore, we characterized the microstructure of the dormant vegetative meristems in the transgenic apple lines and Japanese apricot cultivars via transmission electron microscopy (TEM). We then determined whether the expression levels of selected target genes are correlated with *PmDAM6* expression in the dormant vegetative buds of Japanese apricot cultivars. We also confirmed the association between phytohormone contents and the vegetative bud dormancy of Japanese apricot cultivars and F_1_ individuals with contrasting dormancy characteristics. The study results elucidated the molecular regulatory effects of PmDAM6 on lipid metabolism, hormone (ABA, CTK, and gibberellin (GA)) biosynthesis and catabolism, and cell division, providing new insights into how Rosaceae DAMs control bud dormancy.

## Results

### Transcriptome analysis using the dormant vegetative buds of *35S:PmDAM6* transgenic apple

We previously produced two independent *PmDAM6*-overexpressing transgenic apple lines (35S-2 and 35S-4), with *PmDAM6* more highly expressed in 35S-4 than in 35S-2 [18]. The results of our phenotypic analyses in the current study were consistent with the previous findings that *PmDAM6*-overexpressing transgenic apple plants have a higher bud set rate (percentage of apices with a terminal bud: WT: 55.9 ± 8.3%, 35S-2: 97.6 ± 3.3%, and 35S-4: 100%) (Supplementary Fig. S1A–B) and a lower bud break competency rate than the WT control under forcing conditions (23°C and 18-h day/6-h night) (Supplementary Fig. S1C). Additionally, their chilling requirement increased by approximately 140 to 180 chill hours (CH) according to the chill hour model [19] and their bud break was delayed (Supplementary Fig. S1C–D), which is in accordance with our earlier results [18] and confirmed that PmDAM6 promotes bud set and represses vegetative bud break. The bud break competency test results suggested November and January correspond to the dormancy establishment and maintenance stages, respectively.

We first identified the genes that were differentially expressed between *35S:PmDAM6* and WT, after which the differentially expressed genes (DEGs) were subjected to a Gene Ontology (GO) enrichment analysis. A total of 3146 genes were expressed at significantly lower levels in the *35S:PmDAM6* dormant buds than in the WT dormant buds in either the dormancy establishment or maintenance stage; the enriched GO terms among these DEGs are presented in Supplementary Fig. S2A–B. There were 457 genes that were consistently down-regulated in *35S:PmDAM6* in both stages (Supplementary Table S1). GO terms such as lipid catabolic process, lignin biosynthetic process, and lignin catabolic process were assigned to the down-regulated DEGs in the dormancy establishment and maintenance stages (Supplementary Fig. S2B). We also identified 685 genes that were expressed at significantly higher levels in the *35S:PmDAM6* dormant buds than in the WT dormant buds in either the dormancy establishment or maintenance stage (Supplementary Fig. S3A). Compared with the corresponding expression in WT, the expression levels of 26 genes were consistently up-regulated in *35S:PmDAM6* in both stages (Supplementary Table S2), including *GA2OX*, which encodes the GA catabolism-related enzyme GA2-oxidase and *DAO*, which encodes the auxin catabolism-related enzyme 2-oxoglutarate-dependent dioxygenase. Notably, the small interfering RNA (siRNA) production-related pathway was enriched among the genes with up-regulated expression levels in *35S:PmDAM6* specifically in the dormancy establishment stage (Supplementary Fig. S3B, Supplementary Table S3), raising the possibility that components of the siRNA production pathway are targeted by PmDAM6, but this will need to be experimentally verified. In contrast, response to heat, response to auxin, response to hypoxia, and gibberellin catabolic process were the enriched GO terms assigned to the up-regulated genes in *35S:PmDAM6* specifically in the dormancy maintenance stage (Supplementary Fig. S3B).

**Figure 1:**
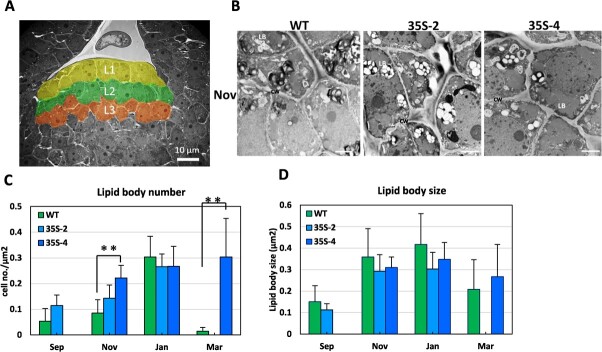
Overexpression of *PmDAM6* resulted in increased lipid body accumulation in the dormant vegetative meristem of transgenic apple plants. Schematic representation of the cells in the L1–L3 layers of the shoot apical meristem that were analyzed for the presence of lipid bodies (A). Transmission electron microscopy images of the L1-L3 layers of dormant meristem (November) of the wild-type (WT) control and two *35S:PmDAM6* lines (35S-2 and 35S-4) (B). LB: lipid body; CW: cell wall. Scale bar = 2 μm. Seasonal changes in the lipid body number (C) and size (D). There were no 35S-4 and 35S-2 buds in September and March, respectively. Data are presented as the mean ± standard error. Significant differences are indicated by ** (*t* test, *P* < 0.01).

### PmDAM6 promotes lipid body accumulation by regulating lipid metabolism-related gene expression in transgenic apple and Japanese apricot

**Figure 2:**
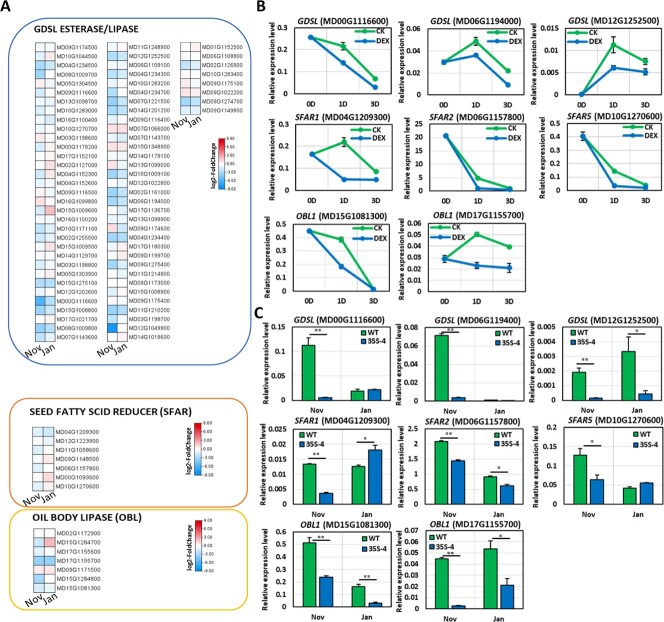
Overexpression of *PmDAM6* repressed the expression of lipid catabolism-related genes in the dormant vegetative buds of transgenic apple plants. Expression heatmaps of the *GDSL ESTERASE/LIPASE*, *SEED FATTY REDUCER* (*SFAR*), and *OIL BODY LIPASE* (*OBL*) genes in the GDDH13 (v1.1) genome (A). The values in the heatmap represent the log_2_(fold-change) in expression [i.e. expression levels in *35S:PmDAM6* (35S-4) transgenic apple compared with the wild-type (WT) expression levels; n = 3]. *GDSL ESTERASE/LIPASE* (MD00G1116600, MD06G1194000, and MD12G1252500), *SFAR* (MD04G1209300, MD06G1157800, and MD10G1270600), and *OBL1* (MD15G1081300 and MD17G1155700) expression in the control and DEX-treated buds of *35S:PmDAM6-GR* plants at 0, 1, and 3 days post-treatment (B). *GDSL ESTERASE/LIPASE* (MD00G1116600, MD06G1194000, and MD12G1252500), *SFAR* (MD04G1209300, MD06G1157800, and MD10G1270600), and *OBL1* (MD15G1081300 and MD17G1155700) expression levels and patterns in the terminal vegetative buds of the WT and 35S-4 plants (C). The values were normalized with the expression level of an apple *SAND* gene. Data are presented as the mean ± standard error (n = 3). Significant differences are indicated by ^**^ or ^*^ (*t* test, *P* < 0.01 or 0.05).

Considering earlier research indicated lipid body metabolism is involved in regulating bud dormancy in poplar [20] and lipid catabolic process was an enriched GO term among the genes that were expressed at significantly lower levels in *35S:PmDAM6* than in WT during the dormancy establishment and maintenance stages in the current study, we examined the accumulation of lipid bodies in the dormant vegetative bud meristem of *35S:PmDAM6*. According to our TEM examination, *35S:PmDAM6* accumulated more lipid bodies per cell than WT, especially during the dormancy establishment stage (Fig. 1). We analyzed the expression of the two key lipid catabolism-related genes *GDSL ESTERASE/LIPASE* and *OIL BODY LIPASE1* (*OBL1*) in *35S:PmDAM6* and WT. In parallel, we assessed the potential effects of the transient induction of DAM functions on candidate gene expression using DEX-induced *PmDAM6*-overexpressing apple plants. Eighty-one of the 87 *GDSL ESTERASE/LIPASE* genes in the GDDH13 (v1.1) apple genome were expressed in the terminal buds (Fig. 2A). The expression levels of 14 *GDSL ESTERASE/LIPASE* genes were consistently lower in *35S:PmDAM6* than in WT in both stages (Fig. 2A). The highly expressed *GDSL ESTERASE/LIPASE* genes (MD00G1116600, MD06G1194000, and MD12G1252500) were all down-regulated in the dormant buds of *35S:PmDAM6-GR* treated with DEX (Fig. 2B). The expression of these genes was consistently down-regulated in 2020/2021 according to the quantitative reverse transcription PCR (qPCR) analysis (Fig. 2C). The *SEED FATTY ACID REDUCER* (*SFAR*) genes, which belong to the *GDSL ESTERASE/LIPASE* family, are regulated by the GA signaling pathway and affect fatty acid storage in seeds [21]. Five of the seven *SFAR* genes in the GDDH13 (v1.1) apple genome were expressed in the buds, with lower expression levels in *35S:PmDAM6* than in WT (Fig. 2A). In addition, the expression of three of these five *SFAR* genes (MD04G1209300, MD06G1157800, and MD10G1270600) was down-regulated by the DEX treatment of *35S:PmDAM6-GR* (Fig. 2B). The qPCR results confirmed the *SFAR* expression levels were consistently lower in *35S:PmDAM6* than in WT (Fig. 2C). This was in accordance with the RNA-seq results. In Arabidopsis, OIL BODY LIPASE1 (OBL1) catabolizes triacylglycerols and modulates lipid body accumulation [22]. Seven of the 10 *OBL* genes were expressed in the buds (Fig. 2A). The expression of five *OBL1* genes (MD15G1081300, MD15G1284700, MD15G1284800, MD17G1155600, and MD17G1155700) was down-regulated in the dormancy establishment stage (Fig. 2A). Additionally, the expression of two *OBL1* genes (MD15G1081300 and MD17G1155700) was down-regulated in *35S:PmDAM6-GR* after the DEX treatment (Fig. 2B). The qPCR results indicated both MD15G1081300 and MD17G1155700 were expressed at lower levels in *35S:PmDAM6* than in WT during the dormancy establishment and maintenance stages (Fig. 2C). We also examined the expression of lipid biosynthesis-related genes. Previous research confirmed lipid droplet-associated protein (LDAP) and lipid droplet-associated protein (LDAP)-interacting protein (LDIP) co-regulate the formation, size, and number of lipid bodies [23–25]. Two of the four *LDAP* genes (MD13G1118800 and MD16G1119000) were expressed in the buds and were more highly expressed in *35S:PmDAM6* than in WT in both stages (Supplementary Table S4), suggesting that PmDAM6 mediates the accumulation of lipid bodies in the dormant vegetative meristem by down-regulating the expression of lipid catabolism-related genes and up-regulating the expression of the genes associated with lipid biosynthesis.

**Figure 3:**
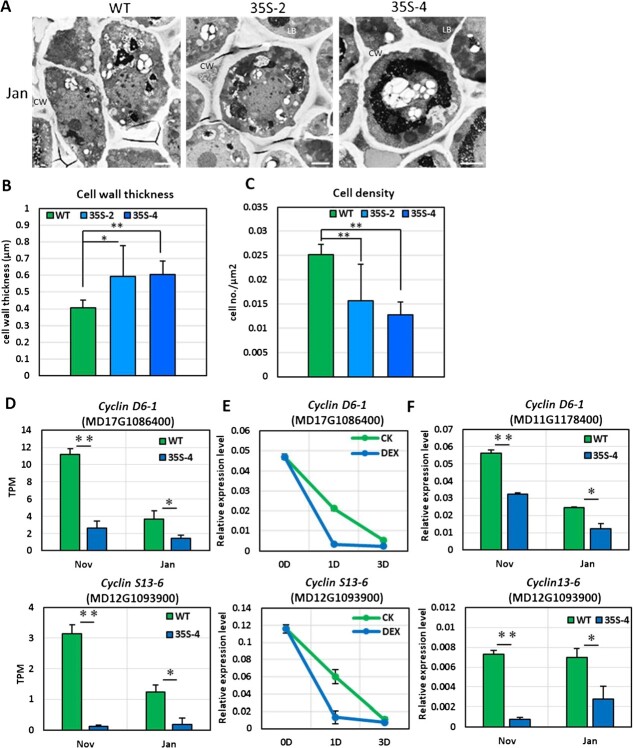
Overexpression of *PmDAM6* inhibited cell division and repressed the expression of cell cycle-related genes in the dormant vegetative meristem of transgenic apple plants. Transmission electron microscopy images of the dormant meristem (January) of the wild-type (WT) control and two *35S:PmDAM6* lines (35S-2 and 35S-4). LB, lipid body; CW, cell wall. Scale bar = 2 μm (A). Cell wall thickness (B) and cell density (C) of the terminal buds collected in January. Data are presented as the mean ± standard error. Significant differences are indicated by ** or * (*t* test, *P* < 0.01 or 0.05). *G1S-SPECIFIC CYCLIN* (MD17G1086400) and *G2 MITOTIC-SPECIFIC CYCLIN* (MD12G1093900) expression levels and patterns in the terminal vegetative buds of WT and *35S:PmDAM6* (35S-4) plants (D) and in the control and DEX-treated buds of *35S:PmDAM6-GR* plants at 0, 1, and 3 days post-treatment (E). *G1S-SPECIFIC CYCLIN* (MD17G1086400) and *G2 MITOTIC-SPECIFIC CYCLIN* (MD12G1093900) expression levels and patterns in the terminal vegetative buds of WT and *35S:PmDAM6* (35S-4) plants in 2020/2021 (F). The values were normalized with the expression level of an apple *SAND* gene. Data are presented as the mean ± standard error (n = 3). Significant differences are indicated by ^**^ or ^*^ (*t* test, *P* < 0.01 or 0.05).

We then examined the relationship between our identified DAM-regulated genes and the reported dormancy-related candidate genes. There was a significant overlap between 5409 genes whose expression levels were highly correlated (|r| ≥ 0.6) with chilling accumulation (Supplementary Fig. S4) in apple [26] and 3750 DEGs in apple (*P* < 0.01). Among these, there were 869 genes that were found to be overlapped (Supplementary Table S5). Many of the 869 significantly overlapping genes were associated with lipid catabolism, including *GDSL ESTERASE/LIPASE* and *SFAR* genes (e.g., *MdSFAR1*, MD04G1209300). The expression of *MdSFAR1* was highly negatively correlated (−0.84) with chilling accumulation in apple (Supplementary Table S5). These results suggest that PmDAM6 controls apple bud dormancy by regulating the lipid metabolic pathway.

The above-mentioned analyses identified several candidate lipid metabolism-related apple genes targeted by PmDAM6. To clarify the relationship between the expression of *PmDAM6* and the expression of these genes in Japanese apricot dormant buds, we conducted a gene expression analysis using the dormant buds of two Japanese apricot cultivars with contrasting dormancy characteristics. For the ‘Ellching’ (low-chill) and ‘Nanko’ (high-chill) Japanese apricot cultivars, vegetative bud break under field conditions occurred in January and March, respectively. The chilling requirement for vegetative bud break was lower for ‘Ellching’ than for ‘Nanko’ (‘Ellching’: 511 CH; ‘Nanko’: 726 CH) (Supplementary Fig. S5A–B). The *PmDAM1–6* expression levels were higher in ‘Nanko’ than in ‘Ellching’ (Supplementary Fig. S5C), which is consistent with our previous findings [17].

Our TEM images of ‘Nanko’ and ‘Ellching’ Japanese apricot vegetative buds indicated that lipid bodies accumulated more in ‘Nanko’ than in ‘Ellching’ in January (Supplementary Fig. S6A–B). Our RNA-seq analysis showed that the expression levels of *GDSL ESTERASE/LIPASE* and *SFAR* orthologs were up-regulated when *PmDAM6* expression decreased in both Japanese apricot cultivars during the dormancy release stage (Supplementary Fig. S6C–E).

### PmDAM6 restricts cell division by down-regulating cell cycle-related gene expression in transgenic apple

Our TEM examination also indicated that the apple *35S:PmDAM6* dormant vegetative meristem had a significantly lower cell density and thicker cell wall than the WT dormant vegetative meristem (WT, 0.025 ± 0.002 cells/μm^2^; 35S-2, 0.016 ± 0.008 cells/μm^2^; 35S-4, 0.013 ± 0.003 cells/μm^2^) (Fig. 3A–C). Hence, we speculated that PmDAM6 may repress cell division. Indeed, the 457 down-regulated genes in the *35S:PmDAM6* dormant buds (relative to the corresponding expression in the WT dormant buds) in the dormancy establishment and maintenance stages (Supplementary Fig. S2A) included two *CYCLIN* genes, *G1S-SPECIFIC CYCLIN D6-1* (*MdCYCD6-1*, MD17G1086400) and *G2 MITOTIC-SPECIFIC CYCLIN S13-6* (*MdCYCS13-6*, MD12G1093900) (Fig. 3D, Supplementary Table S1). Furthermore, *MdCYCD6-1* and *MdCYCS13-6* expression levels were down-regulated in the DEX-treated *35S:PmDAM6-GR* (Fig. 3E). The *MdCYCD6-1* and *MdCYCS13-6* expression levels were consistently lower in the *35S:PmDAM6* transgenic apple than in the WT control in both stages according to the qPCR analysis (Fig. 3F).

We also analyzed *CYCLIN* expression and cell density in the shoot apical meristem of Japanese apricot vegetative buds. Two *MdCYCD6-1* orthologs (LOC103329497 and LOC103324617) and three *MdCYCS13-6* orthologs (LOC103319490, LOC103319481, and LOC103326905) are present in Japanese apricot (Supplementary Fig. S7A–B). The expression levels of *PmCYCD6-1* (LOC103329497) and three *PmCYCS13-6* genes (LOC103319490, LOC103319481, and LOC103326905) were negatively correlated with *PmDAM6* expression in both ‘Nanko’ and ‘Ellching’ (Supplementary Fig. S7), whereas the cell density did not differ significantly between ‘Nanko’ and ‘Ellching’ (data not shown). In addition, the plasmodesmata sphincter, which is a typical morphological characteristic of dormancy in poplar [27], was undetectable in our TEM images of the L1–L3 cell layers of the vegetative meristem in our tested samples (data not shown).

### PmDAM6 regulates ABA, CTK, and GA metabolism-related gene expression in transgenic apple

We previously reported that the overexpression of *PmDAM6* increases and decreases the accumulation of ABA and CTK, respectively, in the dormant buds of apple plants [18]; however, the molecular mechanism through which PmDAM6 regulates these changes in phytohormone accumulation is unclear. Thus, we analyzed the expression of genes related to phytohormone biosynthesis and catabolism in *PmDAM6*-overexpressing apple plants.

**Figure 4:**
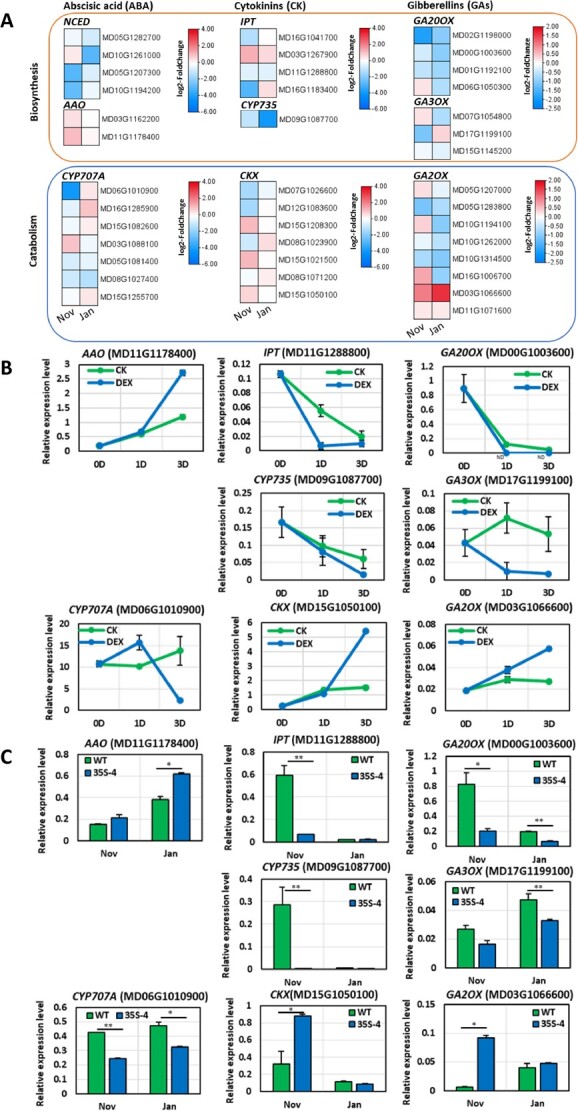
Overexpression of *PmDAM6* modified ABA, CTK, and GA metabolism-related gene expression in apple dormant vegetative buds. Expression heatmaps of the ABA, CTK, and GA biosynthesis and catabolism genes in the GDDH13 (v1.1) genome (A). The values in the heatmap represent the log_2_(fold-change) in expression [i.e. expression levels in *35S:PmDAM6* (35S-4) transgenic apple compared with the wild-type (WT) expression levels; n = 3]. ND, not detected. *AAO3* (MD11G1178400), *CYP707A* (MD06G1010900), *IPT* (MD11G1288800), *CYP735* (MD09G1087700), *CKX* (MD15G1050100), *GA20OX* (MD00G1003500), *GA3OX* (MD17G1199100), and *GA2OX* (MD03G1066600) expression in the control and DEX-treated buds of *35S:PmDAM6-GR* plants at 0, 1, and 3 days post-treatment (B). *AAO3* (MD11G1178400), *CYP707A* (MD06G1010900), *IPT* (MD11G1288800), *CYP735* (MD09G1087700), *CKX* (MD15G1050100), *GA20OX* (MD00G1003500), *GA3OX* (MD17G1199100), and *GA2OX* (MD03G1066600) expression in the terminal vegetative buds of WT and *35S:PmDAM6* (35S-4) plants (C). The values were normalized with the expression level of an apple *SAND* gene. Data are presented as the mean ± standard error (n = 3). Significant differences are indicated by ^**^ or ^*^ (*t* test, *P* < 0.01 or 0.05).

According to the RNA-seq analysis, among the ABA-related genes, the expression levels of the ABA biosynthesis-related *ARABIDOPSIS ALDEHYDE OXIDASE 3* (*MdAAO3*) genes (MD03G1162200 and MD11G1178400) were significantly up-regulated in *35S:PmDAM6* (Fig. 4A) during the dormancy establishment stage. Moreover, *MdAAO3* (MD11G1178400) expression was significantly up-regulated in *35S:PmDAM6-GR* in response to the DEX treatment (Fig. 4B). The qPCR data confirmed the increased expression of *MdAAO3* (MD11G1178400) in *35S:PmDAM6* (Fig. 4C), suggesting that PmDAM6 may promote *MdAAO3* expression during the dormancy establishment stage. In Japanese pear (*Pyrus pyrifolia* Nakai) and apple, DAMs regulate *9-cis-EPOXYCAROTENOID DIOXYGENASE* (*NCED*) expression levels [15, 16, 28]. We identified five *NCED* orthologs in the GDDH13 (v1.1) apple genome, of which *MdNCED3-1* and *MdNCED3-2* (MD05G1207300 and MD10G1194200) and *MdNCED5-1* and *MdNCED5-2* (MD05G1282700 and MD10G1261000) were expressed in bud tissues. The *MdNCED3-1*, *MdNCED3-2*, and *MdNCED5-1* expression levels were lower in *35S:PmDAM6* than in WT in November (Fig. 3A). Only *MdNCED5-2*, which was the *NCED* gene with the lowest expression level in the buds, was expressed at a higher level in *35S:PmDAM6* than in WT (Fig. 4A). A total of 11 *CYTOCHROME P707A* (*CYP707A*) genes, which are key ABA catabolism-related genes, were identified in the GDDH13 (v1.1) apple genome. Of the seven *CYP707A* genes expressed in the buds, the most highly expressed gene (MD06G1010900) was expressed at a lower level in *35S:PmDAM6* than in WT. Notably, MD06G1010900 expression was initially up-regulated and then down-regulated in *35S:PmDAM6-GR* after the DEX treatment (Fig. 4A–B).

Among the genes associated with CTK biosynthesis, *ISOPENTENYL TRANSFERASE* (*IPT*) and *CYP735A* encode proteins that catalyze the biosynthesis of isopentenyladenine (iP) and trans-zeatin (tZ) [29, 30]. We observed that one *IPT* gene (MD11G1288800) and one *CYP735A* gene (MD09G1087700) were expressed at lower levels in *35S:PmDAM6* than in WT in the dormancy establishment and maintenance stages (Fig. 4A). In the GR line, the expression of *MdIPT* (MD11G1288800) and *MdCYP735A* (MD09G1087700) was suppressed after the DEX treatment (Fig. 4B). The qPCR results also revealed the inhibitory effects of the overexpression of *PmDAM6* on *IPT* and *CYP735A* expression (Fig. 4C). Conversely, the expression levels of four of seven *CYTOKININ DEHYDROGENASE* (*CKX*) genes were higher in *35S:PmDAM6* than in WT, including *MdCKX* (MD15G1050100), which was the most highly expressed *MdCKX* gene (Fig. 4A). Moreover, *MdCKX* (MD15G1050100) expression was up-regulated in the DEX-treated *35S:PmDAM6-GR* samples (Fig. 4B). The qPCR analysis confirmed that the overexpression of *PmDAM6* up-regulated *MdCKX* (MD15G1050100) expression (Fig. 4C).

In woody perennials, pathways regulating GA metabolism are important for dormancy induction and release [31, 32]. Thus, we examined GA metabolism-related gene expression in *35S:PmDAM6* apple plants. Five GA catabolism-related *MdGA2OX* genes (MD5G1207000, MD10G1194100, MD16G1006700, MD03G1066600, and MD11G1071600) were more highly expressed in *35S:PmDAM6* than in WT in November (Fig. 4A). Additionally, *MdGA2OX* (MD03G1066600) expression was up-regulated in *35S:PmDAM6-GR* following the DEX treatment (Fig. 4B). These results were consistent with the qPCR data (Fig. 4C). The analysis of 869 chilling-related DEGs indicated the expression of *MdGA2OX* (MD03G1066600) was highly negatively correlated (−0.79) with chilling accumulation in apple (Supplementary Table S5).

In terms of the GA biosynthesis-related genes, the *MdGA20OX* (MD02G1198000, MD00G1003600, and MD01G1192100) expression levels were lower in *35S:PmDAM6* than in WT in the dormancy establishment and maintenance stages (Fig. 4A). Moreover, the expression levels of *MdGA3OX* (MD17G1199100, MD15G1145200, and MD07G1054800) were lower in *35S:PmDAM6* than in WT in either the dormancy establishment or maintenance stage (Fig. 4A). Furthermore, *MdGA20OX* (MD00G1003600) expression was undetectable in *35S:PmDAM6-GR* at 1 and 3 days after the DEX treatment (Fig. 4B). The expression of *MdGA3OX* (MD17G1199100) was down-regulated in *35S:PmDAM6-GR* treated with DEX (Fig. 4B). The *MdGA20OX* (MD00G1003600) and *MdGA3OX* (MD17G1199100) expression levels were also consistently down-regulated in *35S:PmDAM6* according to the qPCR analysis (Fig. 4C).

### RNA-seq analysis revealed the correlation between *PmDAM6* expression and ABA, GA, and CTK metabolism-related gene expression in Japanese apricot

**Figure 5:**
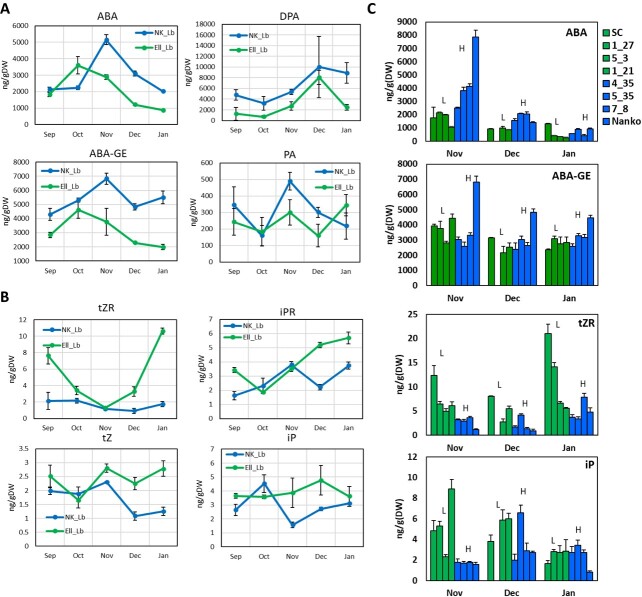
Seasonal changes in abscisic acid and cytokinin metabolite levels in the vegetative buds of Japanese apricot cultivars with contrasting chilling requirements for bud break and F_1_ individuals. Seasonal accumulation of abscisic acid (ABA), abscisic acid glucosyl ester (ABA-GE), phaseic acid (PA), and dihydrophaseic acid (DPA) in the dormant vegetative buds of high-chill ‘Nanko’ and low-chill ‘Ellching’ (A). Seasonal accumulation of trans-zeatin riboside (tZR), trans-zeatin (tZ), isopentenyladenine (iP), and isopentenyladenine riboside (iPR) in the vegetative buds of high-chill ‘Nanko’ and low-chill ‘Ellching’ (B). Seasonal accumulation of ABA, ABA-GE, tZR, and iP in the vegetative buds of F_1_ individuals with contrasting chilling requirements: low-chill type (L: SC, 1_27, 5_3, 1_21 ) and high-chill type (H: 4_35, 5_35, 7_8, Nanko). Data are presented as the mean ± standard error (n = 3).

We first quantified the ABA and CTK contents in the dormant buds of the two Japanese apricot cultivars with contrasting chilling requirements and their F_1_ offspring. The ABA and ABA-related catabolite [ABA-glucosyl ester (ABA-GE), phaseic acid (PA), and dihydrophaseic acid (DPA)] contents were lower in ‘Ellching’ than in ‘Nanko’ (Fig. 5A). The *PmDAM6* expression profile was more highly correlated with the ABA and ABA-GE levels throughout the dormancy cycle (correlation coefficient = 0.49 and 0.58, respectively) than with the contents of the other analyzed plant hormone metabolites (data not shown). The contents of the active CTK precursors [trans-zeatin riboside (tZR) and isopentenyladenine riboside (iPR)] were higher in ‘Ellching’ than in ‘Nanko’ in most of the analyzed months (Fig. 5B). During the ‘Ellching’ dormancy release stage in December, the active CTKs (tZ and iP) were more abundant in ‘Ellching’ than in ‘Nanko’ (Fig. 5B). The iP content in ‘Nanko’ increased slowly in January, which is when dormancy was released (Fig. 5B). Similarly, in the F_1_ offspring derived from the cross between the high-chill ‘Nanko’ and the low-chill ‘SC’, the ABA and ABA catabolite contents were generally higher in the high-chill individuals than in the low-chill individuals, whereas the CTK and CTK precursor contents were higher in the low-chill individuals than in the high-chill individuals (Fig. 5C).

We then analyzed the *AAO* and CTK metabolism-related gene expression patterns. In both ‘Nanko’ and ‘Ellching’, *PmAAO3* (LOC103330122) expression tended to be down-regulated when the *PmDAM6* expression level decreased during dormancy release (Supplementary Fig. S8A). In both ‘Nanko’ (high-chill) and ‘Ellching’ (low-chill), the *PmIPT* (LOC1033341494) and *PmCYP735A* (LOC103329680) expression levels increased significantly as the *PmDAM6* expression level decreased (Supplementary Fig. S8B–C). Additionally, *PmIPT* (LOC103341494) was expressed at lower levels in ‘Nanko’ than in ‘Ellching’ at all stages (Supplementary Fig. S8B). Two *PmCKX* genes (LOC103330694 and LOC103344279) were expressed at higher levels in ‘Nanko’ than in ‘Ellching’ throughout the dormancy process (Supplementary Fig. S8D), whereas the opposite patterns were observed for the other *PmCKX* genes. Among the GA metabolism-related genes, the expression levels of the GA biosynthesis-related genes, especially the *PmGA3OX* genes (LOC103328005 and LOC103340995), were consistently lower in ‘Nanko’ than in ‘Ellching’ across most stages (Supplementary Fig. S9A). Furthermore, *PmGA3OX* (LOC103328005) expression in both ‘Nanko’ and ‘Ellching’ was substantially up-regulated following a sharp decrease in *PmDAM6* expression (Supplementary Fig. S9B). The *PmGA2OX* expression levels were higher in ‘Nanko’ than in ‘Ellching’ in most stages, with the *PmGA2OX* (LOC103325127) expression trend similar to that of *PmDAM6* in both cultivars (Supplementary Fig. S9C). Moreover, *PmGA20OX* expression levels were higher in ‘Nanko’ than in ‘Ellching’ in most stages (Supplementary Fig. S9A).

## Discussion

The molecular mechanism mediating Rosaceae DAM functions remains unclear. Thus, identifying new DAM targets as well as verifying the effects of DAMs on the previously reported putative targets in bud meristematic tissues may clarify the molecular basis of DAM effects. Accordingly, in this study, we used established genetic materials, including *DAM*-overexpressing transgenic lines and breeding populations comprising high-chill and low-chill cultivars, to elucidate how DAMs affect bud dormancy. More specifically, we combined TEM examinations with analyses of gene expression in the vegetative meristem of *PmDAM6*-overexpressing apple lines and Japanese apricot cultivars with contrasting dormancy traits to clarify how PmDAM6 modifies cellular metabolism in the dormant vegetative meristem.

Notably, we observed that PmDAM6 promotes the accumulation of lipid bodies in dormant vegetative buds. To the best of our knowledge, this is the first report describing how lipid metabolism in the vegetative meristem is involved in the regulation of bud dormancy via SVP/DAM. During dormancy, lipid bodies accumulate in the buds of perennial plants [33, 34]. Lipid body levels reportedly increase in apple vegetative buds during the endodormancy induction stage [35]. Additionally, lipid bodies accumulate in apple floral buds as the chilling exposure increases [36]. In hybrid aspen (*Populus tremula* × *Populus tremuloides*), *LDAP* expression levels increase significantly during the dormancy induction stage [20]. In Arabidopsis, SFARs belonging to the GDSL ESTERASE/LIPASE family inhibit fatty acid storage [21]. Although the GDSL ESTERASE/LIPASE effects on bud dormancy will need to be clarified, increases in *GDSL ESTERASE/LIPASE* expression reportedly enhance seed germination in several species (*Brassica napus*, *Gossypium hirsutum*, and *Nicotiana tabacum*) [37–40]. The overexpression of *SFAR4* in Arabidopsis leads to an increase in the germination rate, but a decrease in the fatty acid content [21, 40]. In addition, OBL1 in Arabidopsis modulates lipid body accumulation [22]. On the basis of our findings in the current study, PmDAM6 may inhibit fatty acid catabolism and enhance the accumulation of lipid bodies by down-regulating the expression of *GDSL ESTERASE/LIPASE*, *SFAR*, and *OBL1* genes.

**Figure 6:**
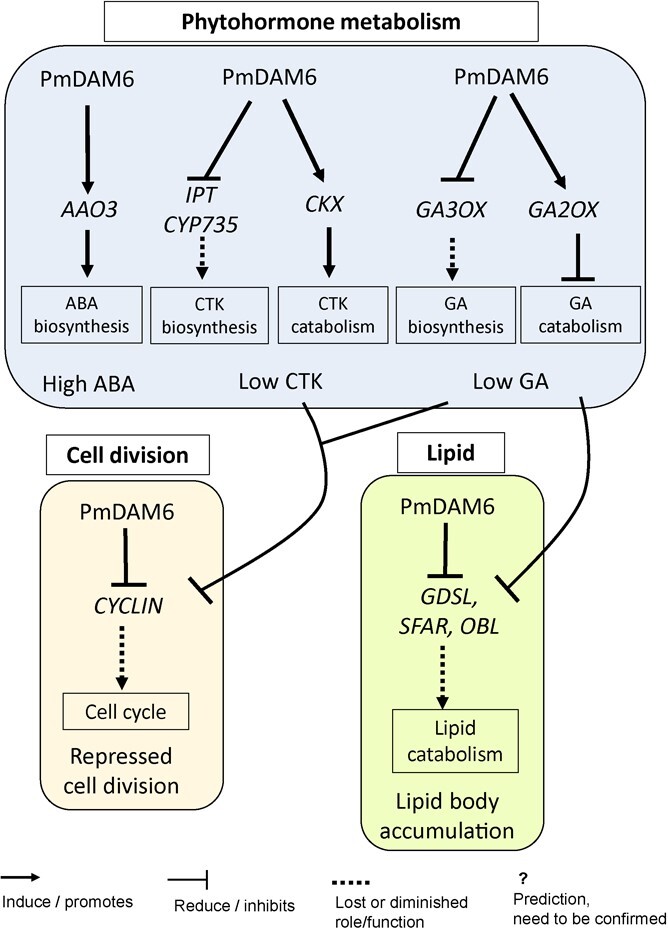
Proposed model illustrating how PmDAM6 regulates ABA, CKT, and GA metabolism, lipid body accumulation, and cell division in the dormant vegetative meristem of *Prunus mume*

In accordance with a previous study that demonstrated the close association between ABA and dormancy depth [14], we observed that the accumulation of ABA and its metabolites is closely correlated with bud dormancy depth and *PmDAM6* expression in the dormant vegetative buds of both transgenic apple lines [18] as well as Japanese apricot cultivars and their F_1_ offspring (this study). After considering the results of our previous research and the data generated in the present study, we propose that PmDAM6 may induce the accumulation of ABA by up-regulating the expression of *AAO3*, which encodes an enzyme that catalyzes the final step of the ABA biosynthesis pathway [41]. Notable effects of *PmDAM6* overexpression were the up-regulation of *GA2OX* and *CKX* expression and the down-regulation of *IPT*, *CYP735A*, and *GA3OX* expression, suggesting that PmDAM6 limits GA and CTK accumulation (Fig. 6). Interestingly, we detected the negative correlation between *GA3OX* and *PmDAM6* expression and the positive correlation between *GA2OX* and *PmDAM6* expression in both transgenic apple lines and two Japanese apricot cultivars (‘Nanko’ and ‘Ellching’). Considered together, our findings imply that PmDAM6 may mediate the decrease in GA accumulation in vegetative buds through the coordinated decrease in GA biosynthesis (via the down-regulated expression of *GA3OX*) and increase in GA catabolism (via the up-regulated expression of *GA2OX*). Additionally, PmDAM6 may mediate the decrease in CTK accumulation by up-regulating *CKX* expression and down-regulating *IPT* and *CYP735A* expression in the dormant vegetative buds of Japanese apricot (Fig. 6; Supplementary Fig. S7B–C). These findings along with the expression-dependent modifications in phytohormone metabolism reflect the repression of cell division. Specifically, the expression levels of the *CYCD* genes related to the G1-to-S-phase transition and *CYCS13–7* encoding a G2/mitotic-specific cyclin, all of which are important for bud outgrowth [42], were down-regulated in the dormant vegetative meristem of the *PmDAM6*-overexpressing plants. In model plants, ABA, GA, and CTK affect cell division, with GA and CTK regulating cell division by modulating *CYCLIN* expression [43–45], whereas ABA induces the expression of *ICK1*, which encodes a cyclin-dependent protein kinase inhibitor that interacts with CYCD3, thereby inhibiting cell division in Arabidopsis [46]. The expression of *CYCLIN* genes is associated with the reactivation of the dormant potato tuber meristem [47]. Accordingly, we propose that PmDAM6 may control dormancy by repressing *CYCLIN* expression either directly or indirectly by decreasing GA and CTK levels (Fig. 6).

Interestingly, increases in GA signaling and an exogenous GA3 treatment of Arabidopsis seeds result in up-regulated *SFAR* expression levels [21]. A similar link between GA and SFAR may exist for the *PmDAM6-*regulated decrease in GA accumulation and *SFAR* expression. Therefore, we speculate that PmDAM6 may promote the accumulation of lipid bodies both directly (by inhibiting lipid catabolism) and indirectly (by inhibiting GA accumulation) (Fig. 6).

## Materials and methods

### Plant materials and growth conditions

This study was completed using transgenic apple (*Malus × domestica*) and Japanese apricot (*P. mume*) plants. We generated the transgenic apple lines (*35S:PmDAM6* and *35S:PmDAM6-GR*) in an earlier study [18]. In the current study, we analyzed two 8-year-old *PmDAM6*-overexpressing (*35S:PmDAM6*) apple lines (35S-2 and 35S-4) in 2019–2020 and 2020–2021 and a 10-year-old DEX-inducible *PmDAM6*-overexpressing (*35S:PmDAM6-GR*) apple line (GR22) in 2021. All lines were grown in a closed greenhouse in Kyoto, Japan under natural photoperiodic conditions. The greenhouse was cooled when the temperature exceeded 25°C (May to September) or 15°C (October to April). The greenhouse was not heated throughout the experimental period. Terminal vegetative buds were collected from WT and 35S-4 plants in November and January in 2019–2020 and 2020–2021, immediately frozen in liquid N_2_, and stored at −80°C prior to the RNA extraction. In November 2021, the terminal vegetative buds of GR22 plants (n = 3–5) were treated with DEX. Briefly, buds were immersed in the 50 μM DEX solution containing 0.1% Tween 20 and 0.2% DMSO or the control solution containing 0.1% Tween 20 and 0.2% DMSO. Buds were incubated at 15°C with shaking at 150 rpm. Buds were collected at 0, 1, and 3 days after the DEX treatment, frozen in liquid N_2_, and stored at −80°C prior to the RNA extraction.

Adult ‘Nanko’ (high-chill) and ‘Ellching’ (low-chill) Japanese apricot trees (>15-years-old) were grown at the Kyoto Experimental Farm of Kyoto University and adult ‘Nanko’ and ‘SC’ (low-chill) trees and their F_1_ offspring, namely three high-chill (4_35, 5_35, and 7_8) and three low-chill (1_27, 5_3, and 1_21) individuals, were grown at the Kizu Experimental Farm. Vegetative buds were collected from ‘Nanko’, ‘Ellching’, ‘SC’, and the F_1_ offspring trees every month from September 2019 to January 2020, immediately frozen in liquid N_2_, and stored at −80°C prior to the RNA extraction and hormone extraction.

The plant materials used for analyses are summarized in Supplementary Table S6.

### Evaluation of the vegetative bud dormancy status of *35S:PmDAM6* apple plants and Japanese apricot cultivars with contrasting chilling requirements and RNA-seq analysis

To investigate the timing of the dormancy phase transition in 2020–2021, bud break competency was tested as previously described [48]. Temperatures were recorded every hour from September 1, 2020, using the TR-50 U2 Thermo Recorder (T&D Corporation, Matsumoto, Japan). In terms of the chill hour model, all hours with temperatures between 0°C and 7.2°C were included [19]. Chilling requirement was considered to be satisfied at the sampling time point when bud break rate became over 40% in forcing condition within 3 weeks. For apple, one-year shoots were collected from WT and transgenic (35S-2 and 35S-4) (n = 9) plants on the 30th of each month from September 2020 to January 2021 as well as on February 28, 2021. The terminal buds were frozen in liquid N_2_ and stored at −80°C. Apple shoots were incubated under forcing conditions (23°C and 18-h day/6-h night). The bud break competency rate was recorded every week until 3 weeks after initiating the incubation. For both Japanese apricot cultivars (‘Nanko’ and ‘Ellching’), shoots were collected on the 15th and 30th of each month from September 2019 to January 2020. The shoots were incubated under forcing conditions (23°C and 18-h day/6-h night). The bud break competency rate was recorded every week until 3 weeks after initiating the incubation.

Total RNA was extracted from the collected buds according to a modified CTAB method [49, 50]. The total RNA was used to construct the RNA-seq library for the 150-bp paired-end sequencing analysis performed using the BGI-SEQ platform (PE150; BGI, Beijing, China). The sequencing analysis for each genotype was completed using three replicates per sample collection time-point.

### Identification of DEGs and GO enrichment analysis

Fastp [51] was used to evaluate the quality of the sequencing data, remove adapters, and eliminate low-quality data. The remaining clean reads were mapped to the *Malus × domestica* GDDH13 (v1.1) genome [52] or to the *P. mume* genome [53] using the default parameters of STAR Aligner [54]. For each sample, a minimum of 10 million clean paired-end reads (on average) were used for the subsequent analysis. The number of reads mapped to each gene was calculated using the Subread package featureCounts program [55]. The raw read counts were normalized and converted to transcripts per million (TPM) values. Published functional annotations of the proteins encoded by the genes in the GDDH13 (v1.1) genome or the *P. mume* genome mapped using the InterProScan database [52] were retrieved. Orthologs were identified using Orthofinder [56]. Only the genes with a TPM value greater than 1 were included in the heatmap. For apple, the genes differentially expressed between the WT and *35S:PmDAM6* plants during dormancy establishment (November) and dormancy maintenance (January) were identified using the DESeq2 R package [57] and the following criteria: |log_2_(fold-change)| > 1 and adjusted *P* < 0.05. A GO enrichment analysis of the DEGs was conducted using the default parameters of the clusterProfiler package (v3.16.1) [58]. The thresholds for identifying significantly enriched GO terms were as follows: pvalueCutoff = 0.01, pAdjustMethod = ‘BH’, and qvalueCutoff = 0.05. We used a previously published RNA-seq dataset for apple buds that underwent a chilling treatment under 5°C for 0, 10, 25, 35, and 65 days, respectively [26]. The correlation coefficient (r) was applied to screen for the genes highly correlated with the chilling unit (CU) (|r| ≥ 0.6). We subsequently examined the DEGs between the WT and *35S:PmDAM6* buds and identified the DEGs that overlapped with the chilling accumulation-correlated genes in apple buds. The overlap between the PmDAM6-associated DEGs and the chilling accumulation-correlated genes was statistically analyzed using Fisher's exact test.

### Expression analysis of selected genes in the buds of *35S:PmDAM6*, WT, and DEX-treated *35S:PmDAM6-GR* apple plants

The expression levels of selected genes were determined by a qPCR analysis. After RNA was extracted from the collected buds as described above, cDNA was synthesized from approximately 1 μg total RNA and then used for the qPCR analysis, which was performed using the THUNDERBIRD SYBR qPCR mix (TOYOBO, Osaka, Japan), gene-specific primers (Supplementary Table S7), and the LightCycler 480 system (Roche, Basel, Switzerland). The apple *SAND* gene (MDP0000185470 and/or MDP0000202305) [59] was selected as the reference control. The PCR program was as follows: 95°C for 30 s and then 45 cycles of 95°C for 15 s, 57°C for 30 s, and 72°C for 60 s. A dissociation curve analysis was performed to confirm that the fluorescence was only derived from gene-specific amplifications. Three replicates were analyzed, with four bud samples per RNA extraction.

### Examination of the apical meristem cells of dormant vegetative buds using a TEM system

Scales were removed from the dormant apple terminal vegetative buds and Japanese apricot lateral vegetative buds. The meristem regions were cut longitudinally in half, fixed in the fixative buffer (2% glutaraldehyde and 4% paraformaldehyde in 0.05 M Na-phosphate buffer, pH 7.2), vacuum infiltrated for 30 min at room temperature, and incubated overnight at 4°C. After the specimens were washed using 0.1 M Na-phosphate buffer (three times for 20 minutes each), they were post-fixed for 2 h in a solution comprising 1% OsO_4_ and 0.1 M Na-phosphate buffer. The specimens were dehydrated using a graded series of ethanol solutions (50%, 60%, 70%, 80%, 90%, and 99%) for 20 minutes each and then in 100% ethanol (two times for 30 min each) and in propylene oxide (two times for 1 h each). The specimens were penetrated by propylene oxide:epon (Luveak 812; Nacalai Tesque, Kyoto, Japan) [1:1 (v/v)] for 1.5 hours, propylene oxide:epon (1:3) for 1.5 hours, and epon only for 12 hours. They were subsequently polymerized using pure epon (60°C) during three overnight incubations and then sliced using a microslicer (DTK-1000; Dosaka EM, Kyoto, Japan) until the apical meristem was visible at the surface of the tissue block. Next, ultra-thin sections (60–80 nm) were prepared using an ultramicrotome (EM UC6; Leica, Heidelberg, Germany). They were then stained with 2% uranyl acetate in 50% ethanol for 20 minutes and then with lead citrate for 2 minutes (Leynolds method). The ultramicrostructure of the apical meristem was examined using a transmission electron microscope (H-7650; Hitachi, Tokyo, Japan). Images were captured using a camera connected to the microscope. Three individual buds were examined for each genotype and time-point. The lipid body size and number per unit area and the cell wall thickness in the L1 to L3 cell layers were calculated using the ImageJ® software (National Institutes of Health, Bethesda, MD, USA). The TEM images for apple, poplar, and *Cunninghamia lanceolata* (Lamb.) Hook generated in previous bud dormancy-related studies [20, 33, 60] were used to identify the lipid bodies in the TEM images generated in this study.

### Examination of the phytohormone contents of Japanese apricot dormant vegetative buds

Vegetative buds were lyophilized and ground to a fine powder using the Multi-beads shocker (Yasui Kikai Co., Osaka, Japan). Abscisic acid and its metabolites as well as CTKs were extracted as previously described [18]. Phytohormone contents were determined by liquid chromatography–triple quadrupole mass spectrometry (Waters; liquid chromatography system: Waters 2695; mass spectrometer: Quattro micro ARI Waters 2996).

## Supplementary Material

Web_Material_uhae102

## Data Availability

All relevant data are provided in the manuscript and its supporting materials. The RNA-seq data for the apple transformants and Japanese apricot generated in this study were deposited in the National Center for Biotechnology Information Gene Expression Omnibus database (http://www.ncbi.nlm.nih.gov/geo) (accession numbers PRJNA960952 and PRJNA958110).
