# Supplementary material for: Regulatory role of Prunus mume DAM6 on lipid body accumulation and phytohormone metabolism in the dormant vegetative meristem
Source: Hortic Res. 2024 Apr 9;11(6):uhae102. doi: 10.1093/hr/uhae102 (PMC11179725; doi:10.1093/hr/uhae102)
Supplement: Web_Material_uhae102 [file web_material_uhae102.zip › 20240315_Supple_Figs ver8.pptx]

## Slide 1
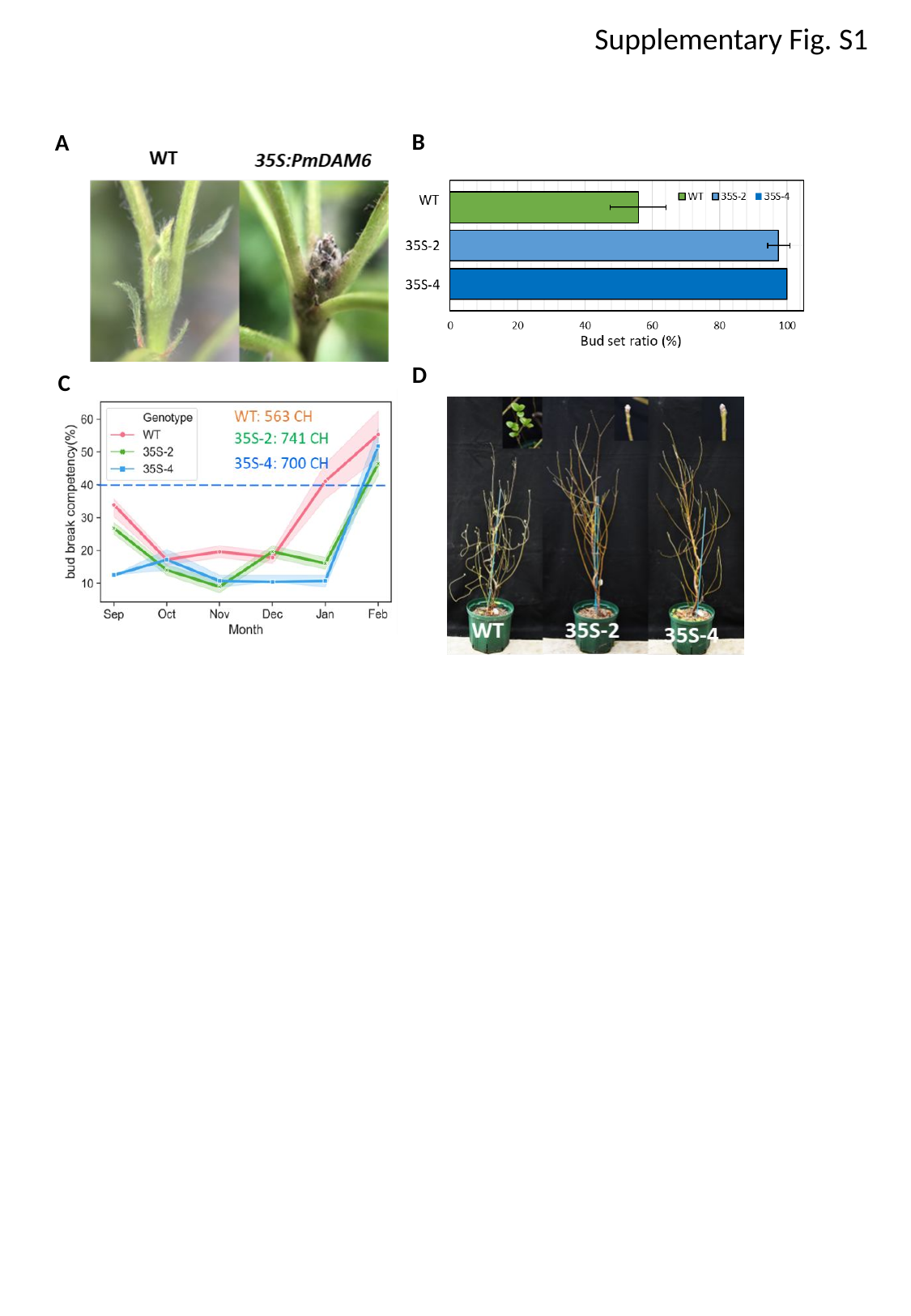

Supplementary Fig. S1
B
A
D
C

## Slide 2
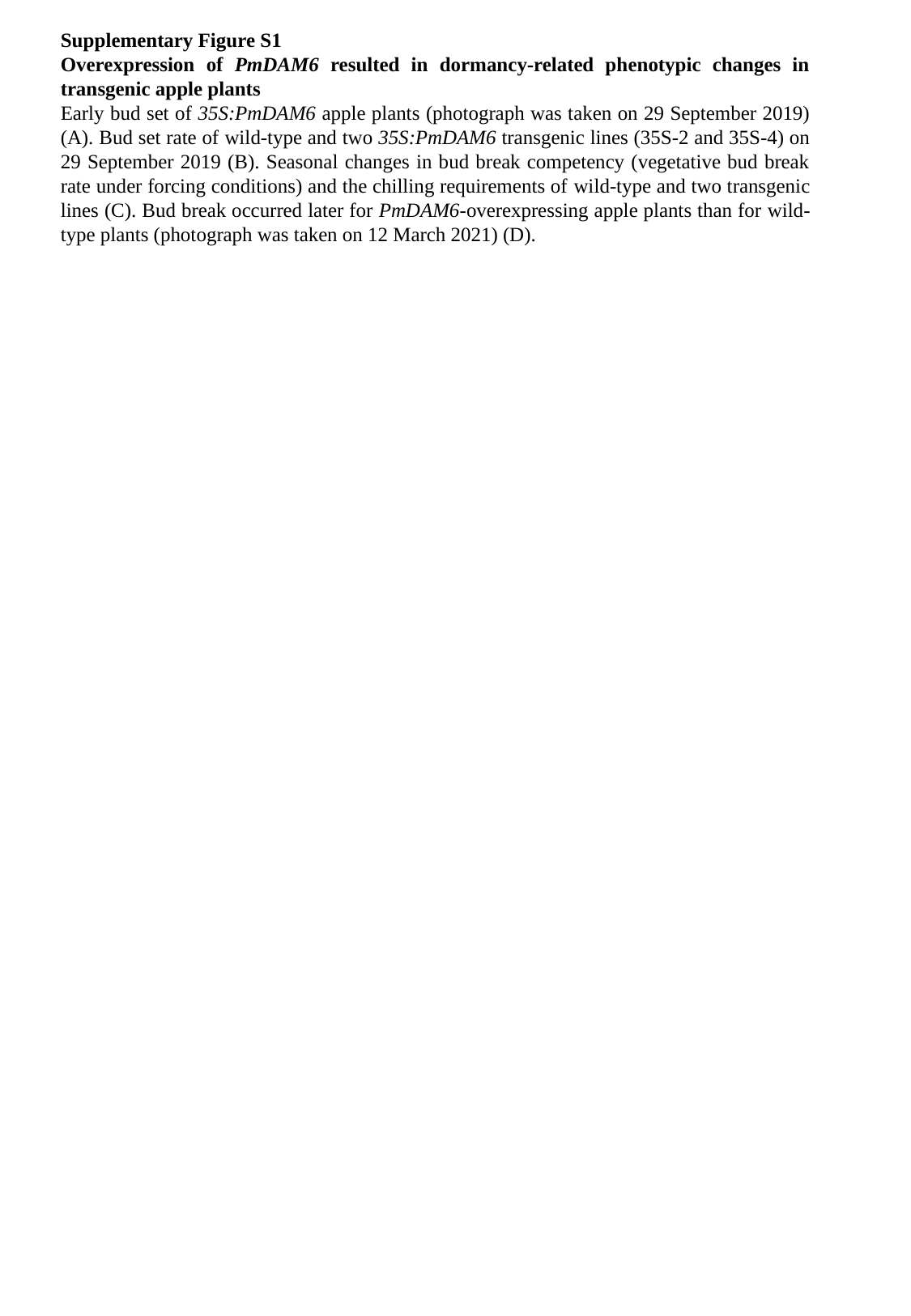

Supplementary Figure S1
Overexpression of PmDAM6 resulted in dormancy-related phenotypic changes in transgenic apple plants
Early bud set of 35S:PmDAM6 apple plants (photograph was taken on 29 September 2019) (A). Bud set rate of wild-type and two 35S:PmDAM6 transgenic lines (35S-2 and 35S-4) on 29 September 2019 (B). Seasonal changes in bud break competency (vegetative bud break rate under forcing conditions) and the chilling requirements of wild-type and two transgenic lines (C). Bud break occurred later for PmDAM6-overexpressing apple plants than for wild-type plants (photograph was taken on 12 March 2021) (D).

## Slide 3
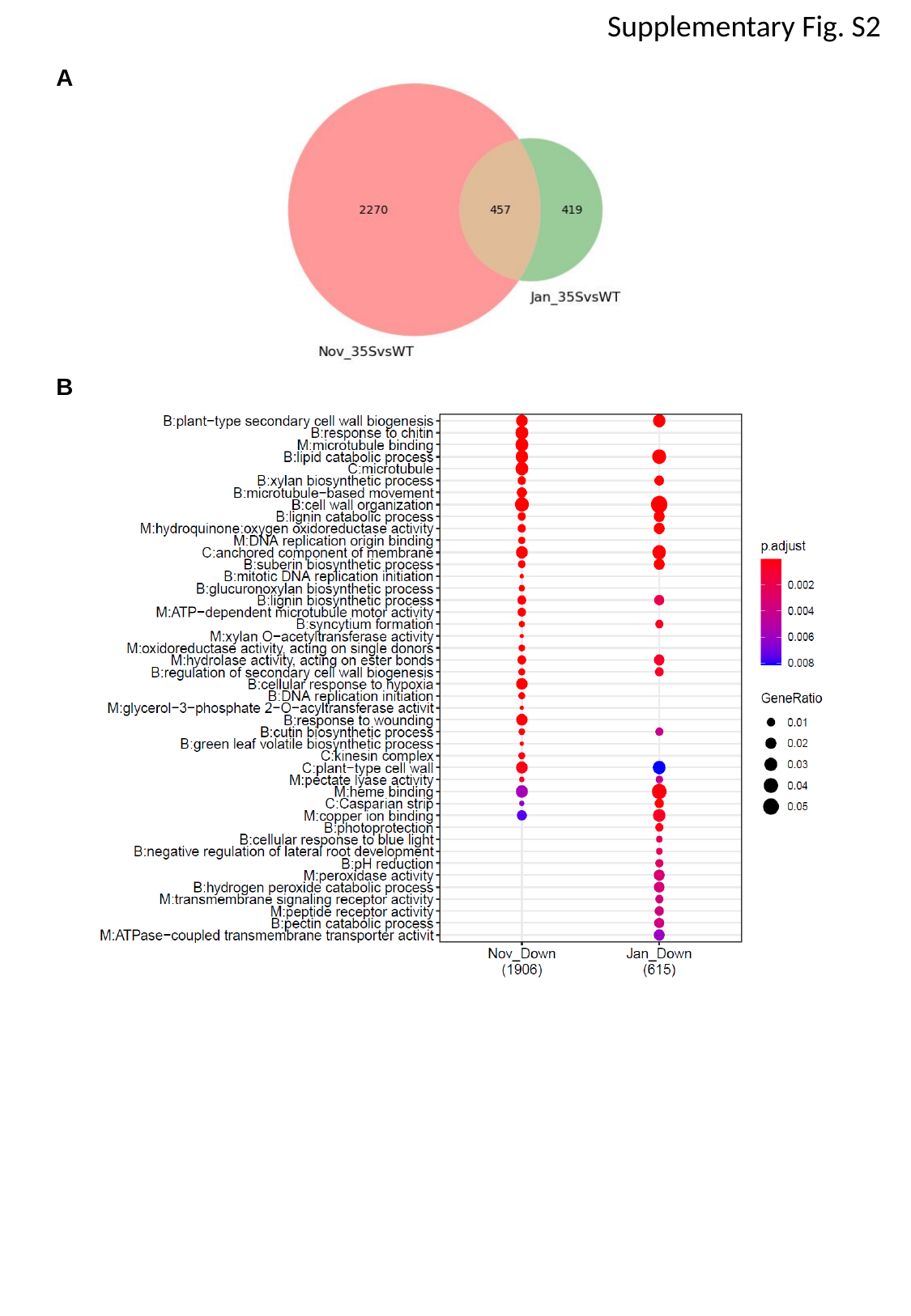

Supplementary Fig. S2
A
B

## Slide 4
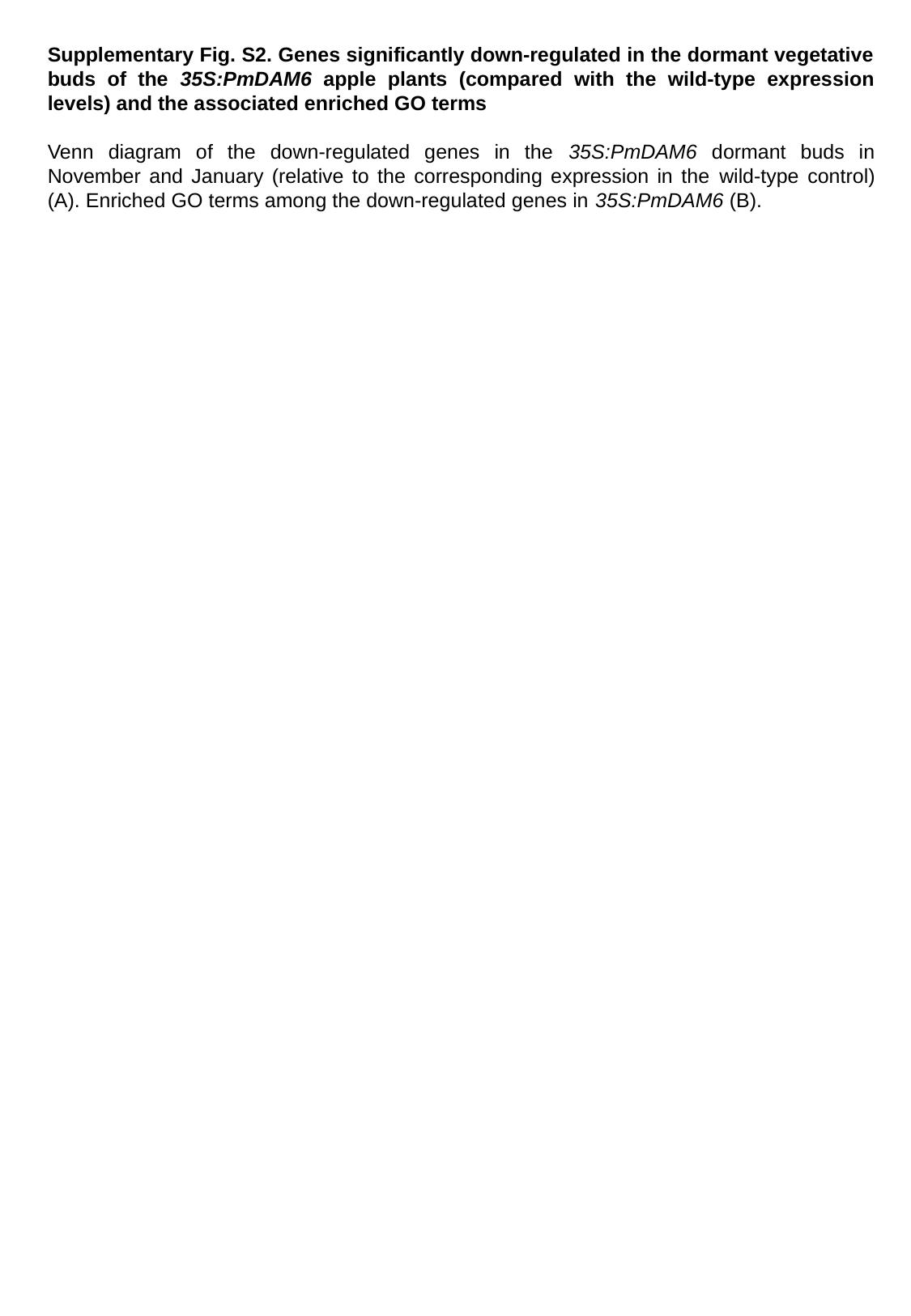

Supplementary Fig. S2. Genes significantly down-regulated in the dormant vegetative buds of the 35S:PmDAM6 apple plants (compared with the wild-type expression levels) and the associated enriched GO terms
Venn diagram of the down-regulated genes in the 35S:PmDAM6 dormant buds in November and January (relative to the corresponding expression in the wild-type control) (A). Enriched GO terms among the down-regulated genes in 35S:PmDAM6 (B).

## Slide 5
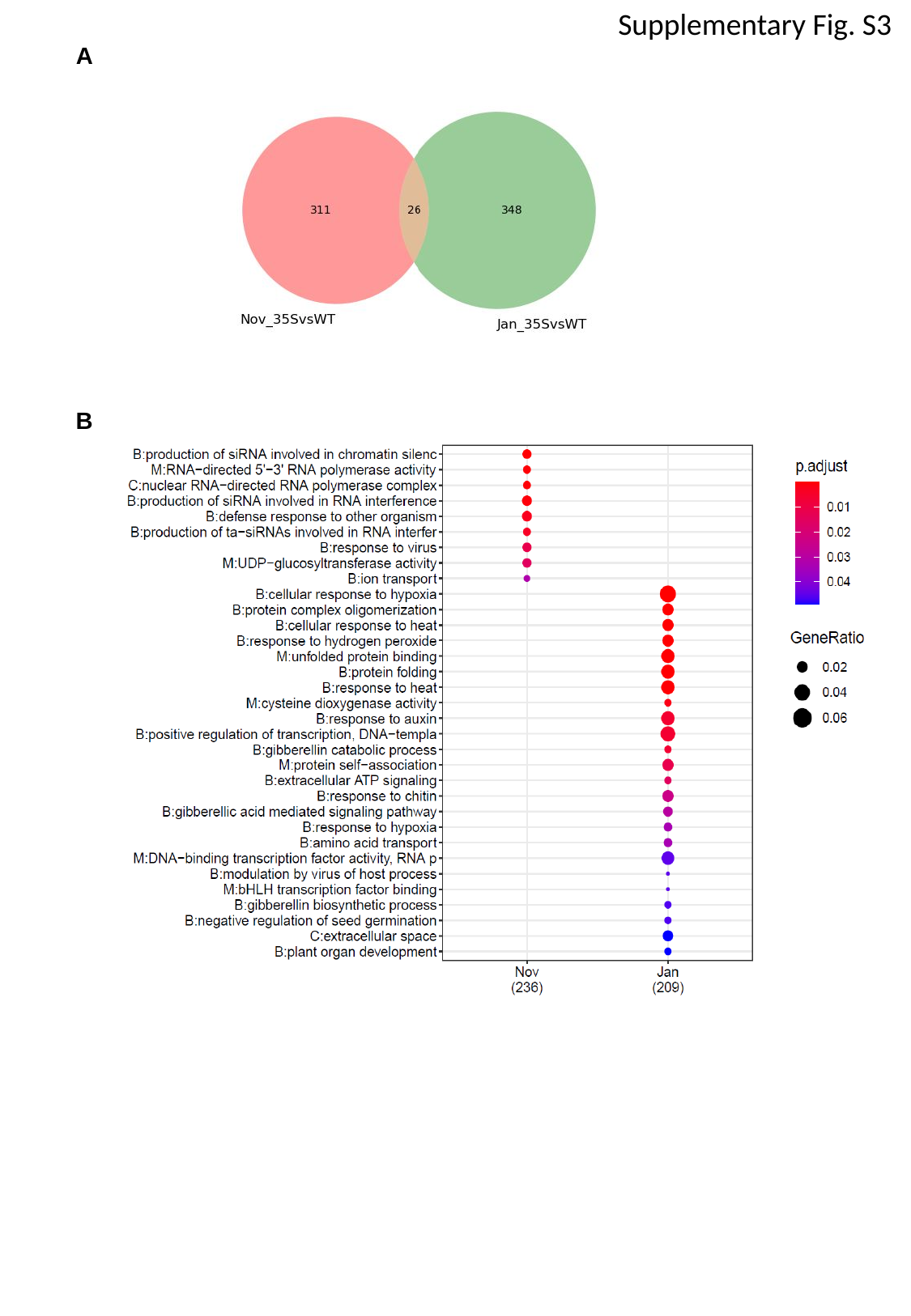

Supplementary Fig. S3
A
B

## Slide 6
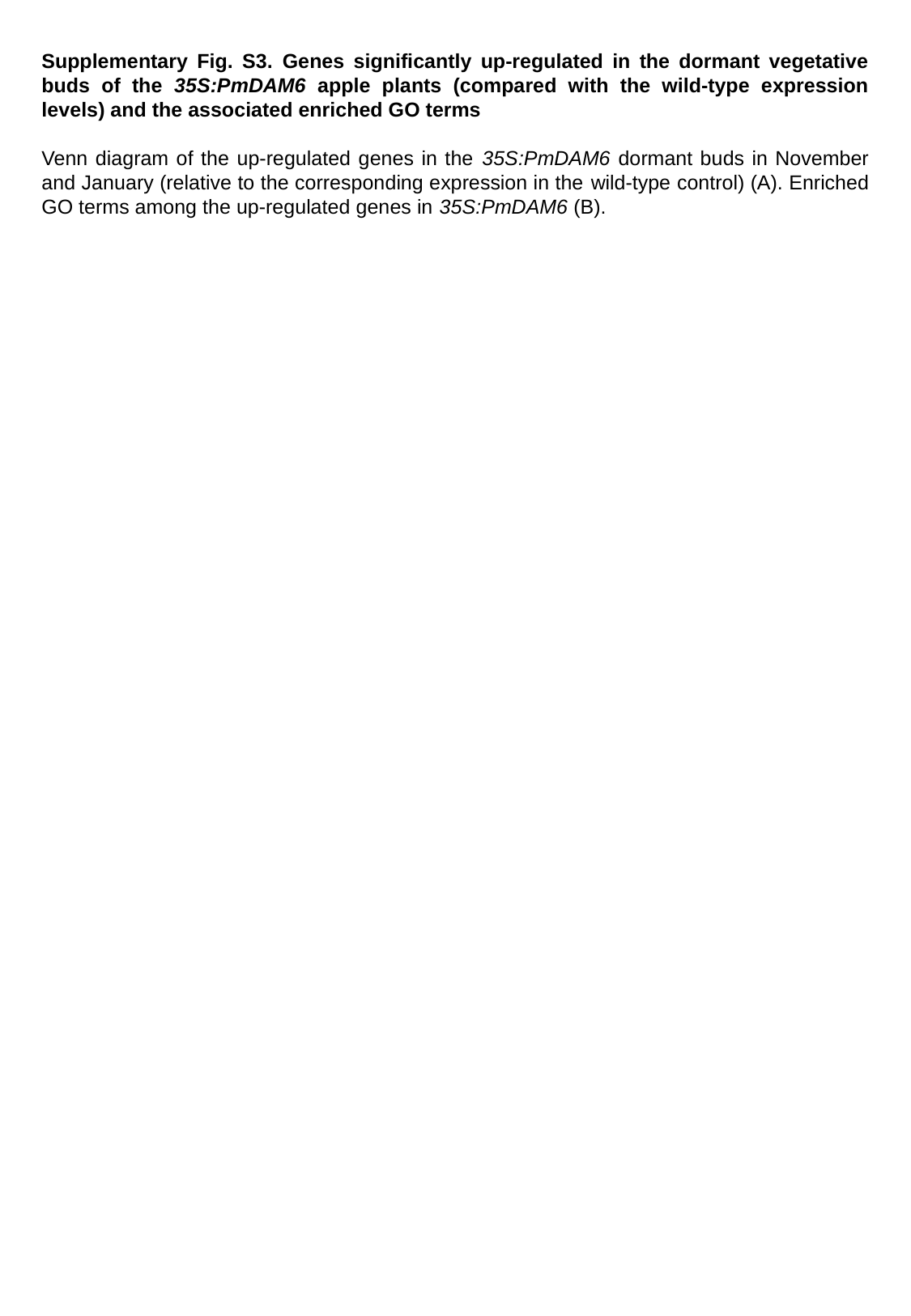

Supplementary Fig. S3. Genes significantly up-regulated in the dormant vegetative buds of the 35S:PmDAM6 apple plants (compared with the wild-type expression levels) and the associated enriched GO terms
Venn diagram of the up-regulated genes in the 35S:PmDAM6 dormant buds in November and January (relative to the corresponding expression in the wild-type control) (A). Enriched GO terms among the up-regulated genes in 35S:PmDAM6 (B).

## Slide 7
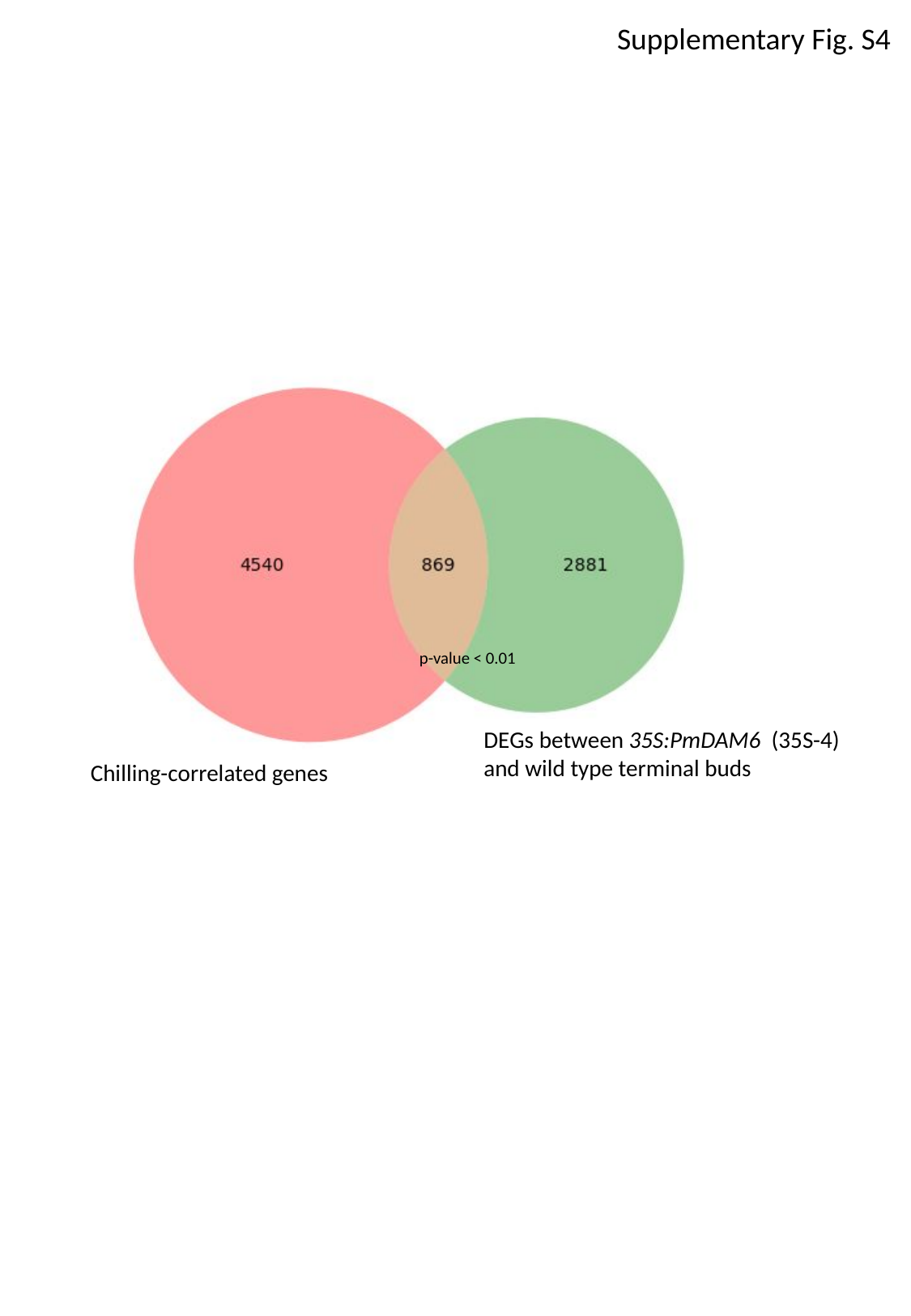

Supplementary Fig. S4
p-value < 0.01
DEGs between 35S:PmDAM6 (35S-4) and wild type terminal buds
Chilling-correlated genes

## Slide 8
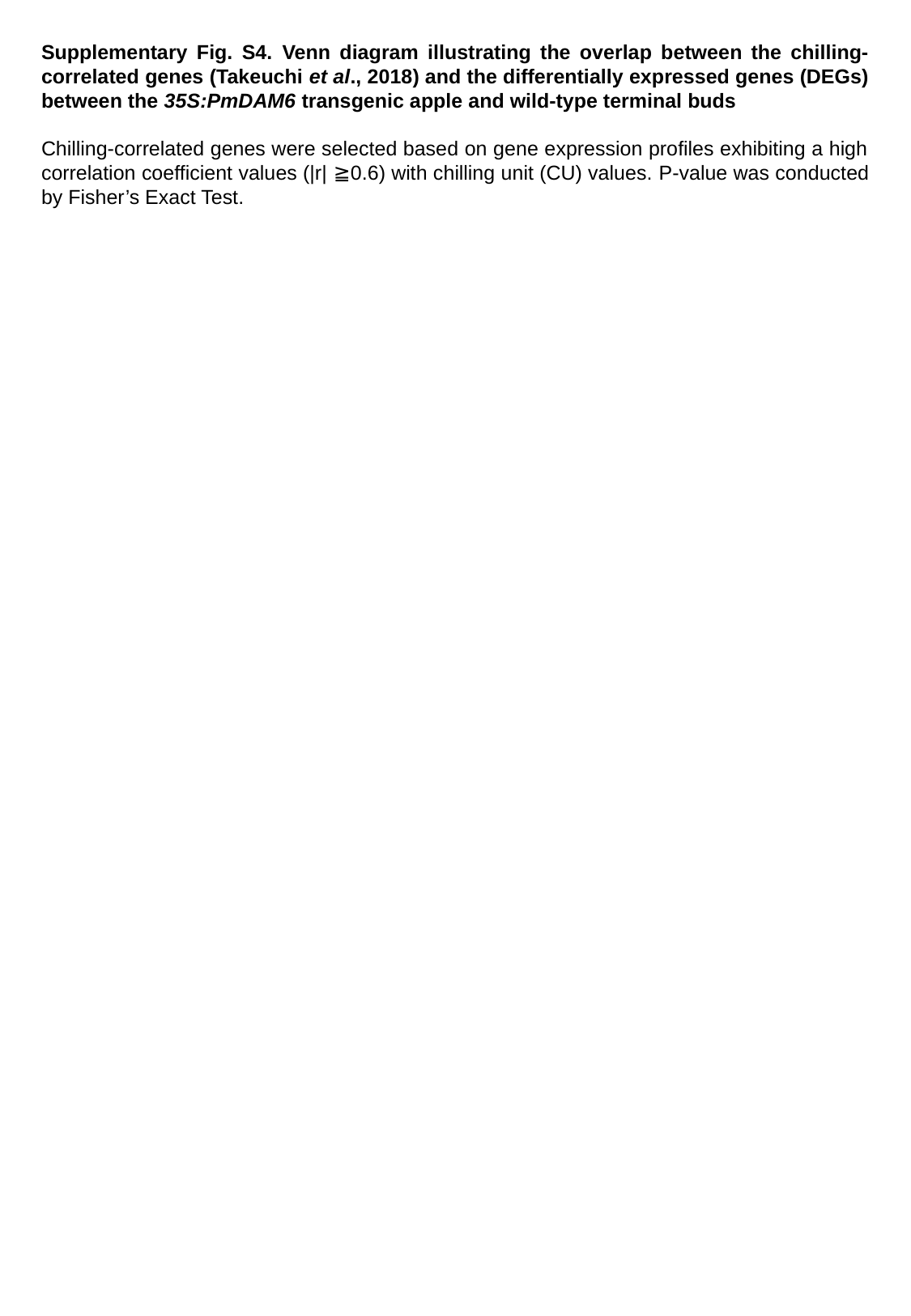

Supplementary Fig. S4. Venn diagram illustrating the overlap between the chilling-correlated genes (Takeuchi et al., 2018) and the differentially expressed genes (DEGs) between the 35S:PmDAM6 transgenic apple and wild-type terminal buds
Chilling-correlated genes were selected based on gene expression profiles exhibiting a high correlation coefficient values (|r| ≧0.6) with chilling unit (CU) values. P-value was conducted by Fisher’s Exact Test.

## Slide 9
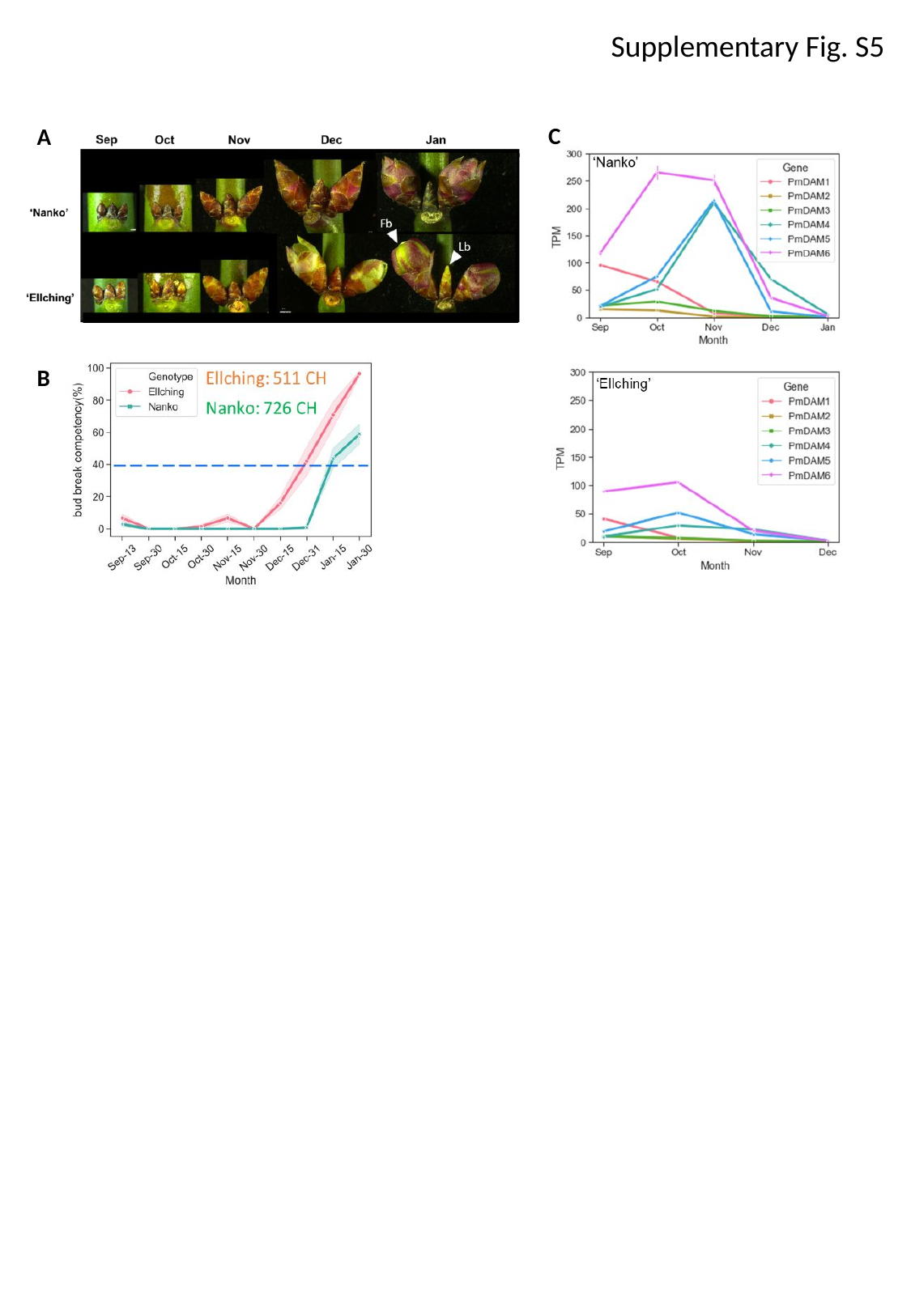

Supplementary Fig. S5
C
A
B

## Slide 10
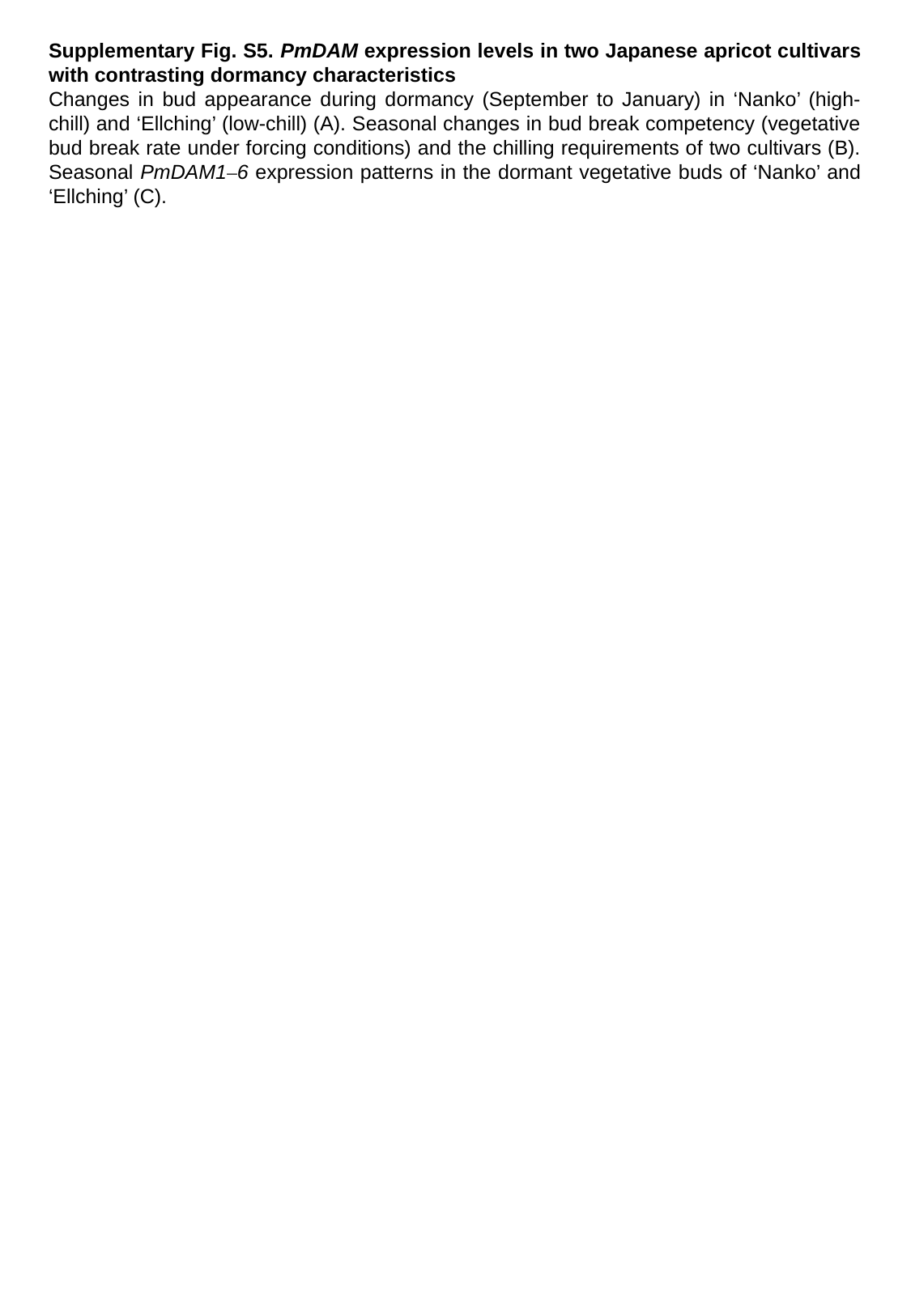

Supplementary Fig. S5. PmDAM expression levels in two Japanese apricot cultivars with contrasting dormancy characteristics
Changes in bud appearance during dormancy (September to January) in ‘Nanko’ (high-chill) and ‘Ellching’ (low-chill) (A). Seasonal changes in bud break competency (vegetative bud break rate under forcing conditions) and the chilling requirements of two cultivars (B). Seasonal PmDAM1–6 expression patterns in the dormant vegetative buds of ‘Nanko’ and ‘Ellching’ (C).

## Slide 11
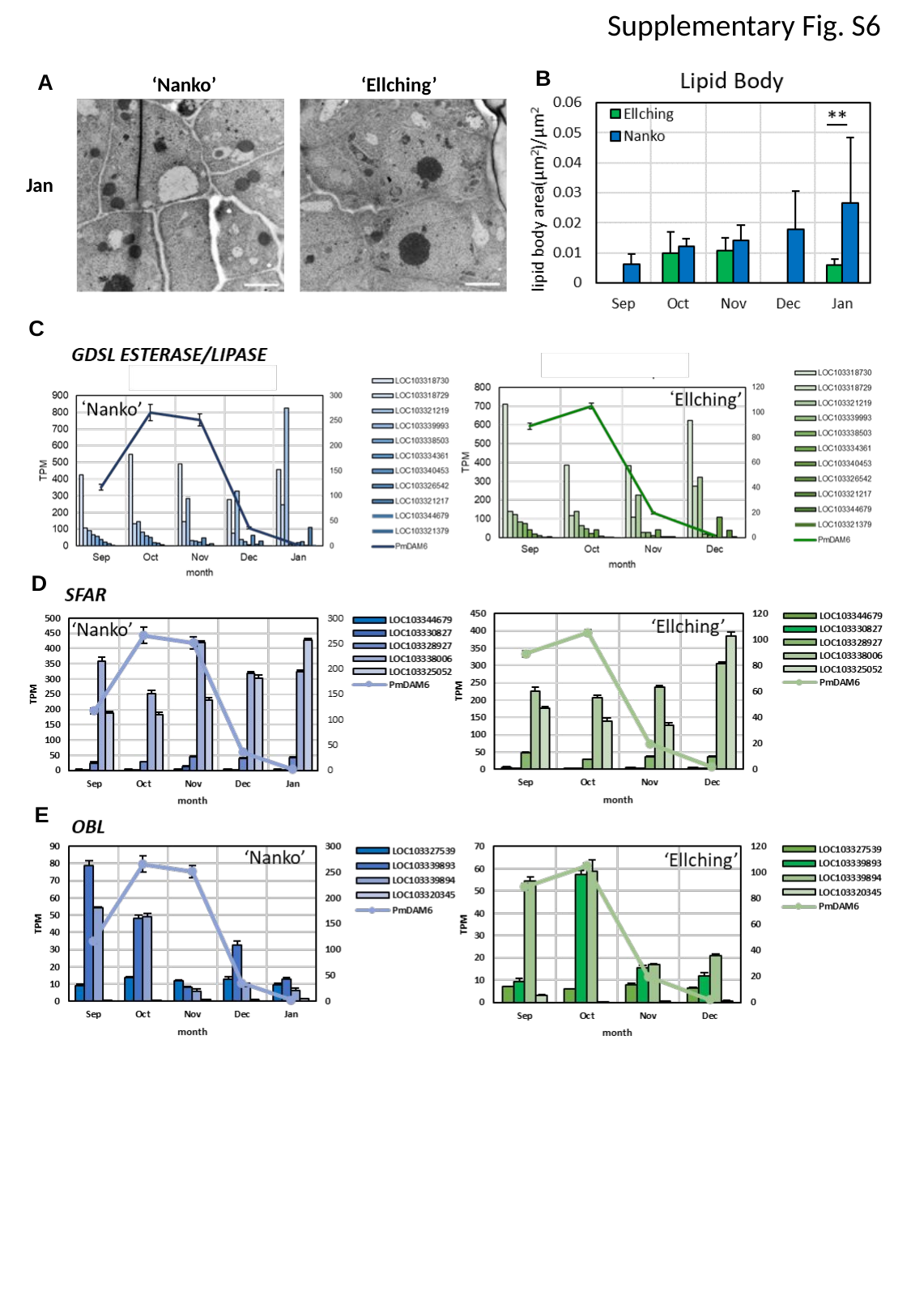

Supplementary Fig. S6
B
A
‘Nanko’
‘Ellching’
Jan
C
D
E

## Slide 12
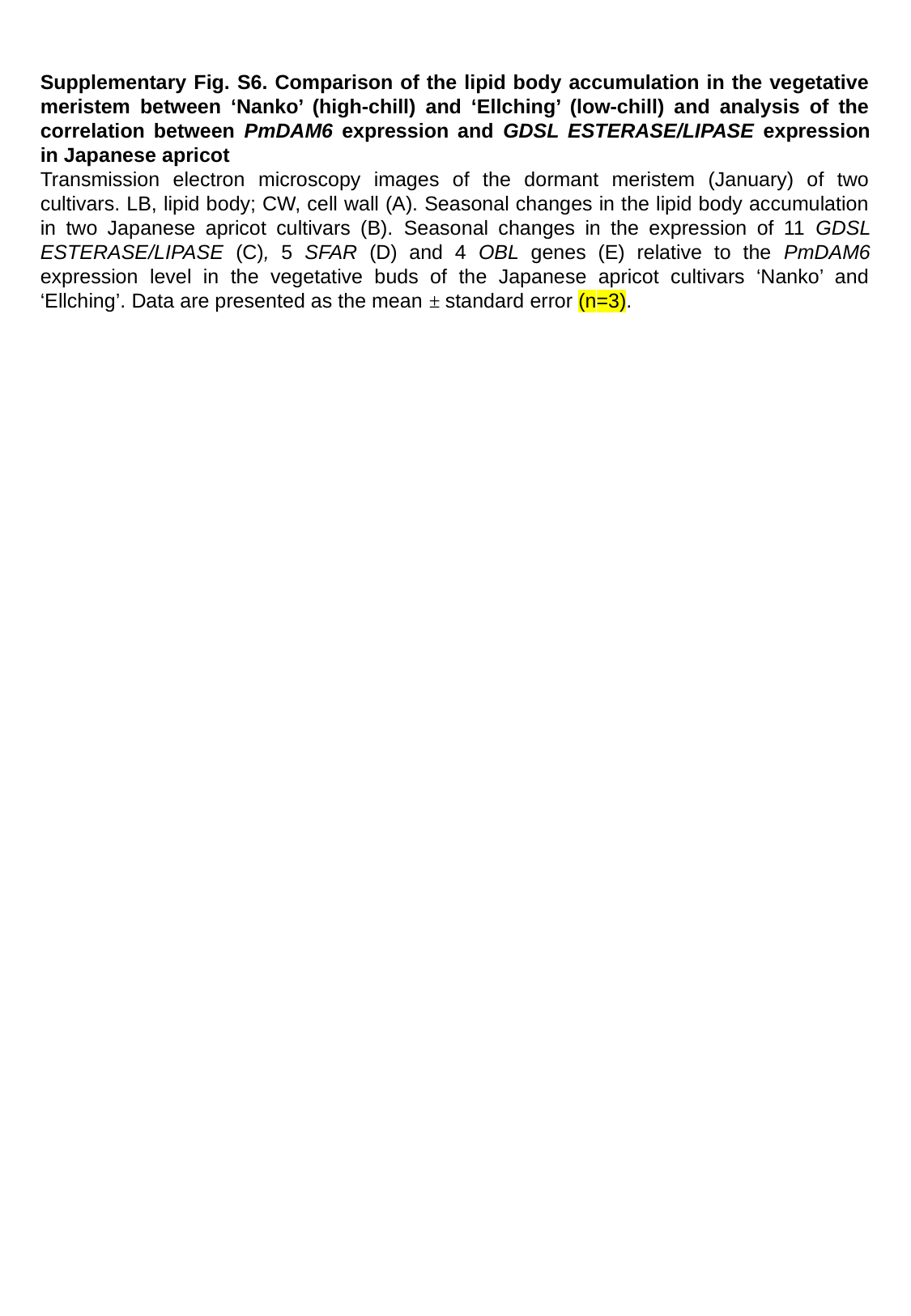

Supplementary Fig. S6. Comparison of the lipid body accumulation in the vegetative meristem between ‘Nanko’ (high-chill) and ‘Ellching’ (low-chill) and analysis of the correlation between PmDAM6 expression and GDSL ESTERASE/LIPASE expression in Japanese apricot
Transmission electron microscopy images of the dormant meristem (January) of two cultivars. LB, lipid body; CW, cell wall (A). Seasonal changes in the lipid body accumulation in two Japanese apricot cultivars (B). Seasonal changes in the expression of 11 GDSL ESTERASE/LIPASE (C), 5 SFAR (D) and 4 OBL genes (E) relative to the PmDAM6 expression level in the vegetative buds of the Japanese apricot cultivars ‘Nanko’ and ‘Ellching’. Data are presented as the mean ± standard error (n=3).

## Slide 13
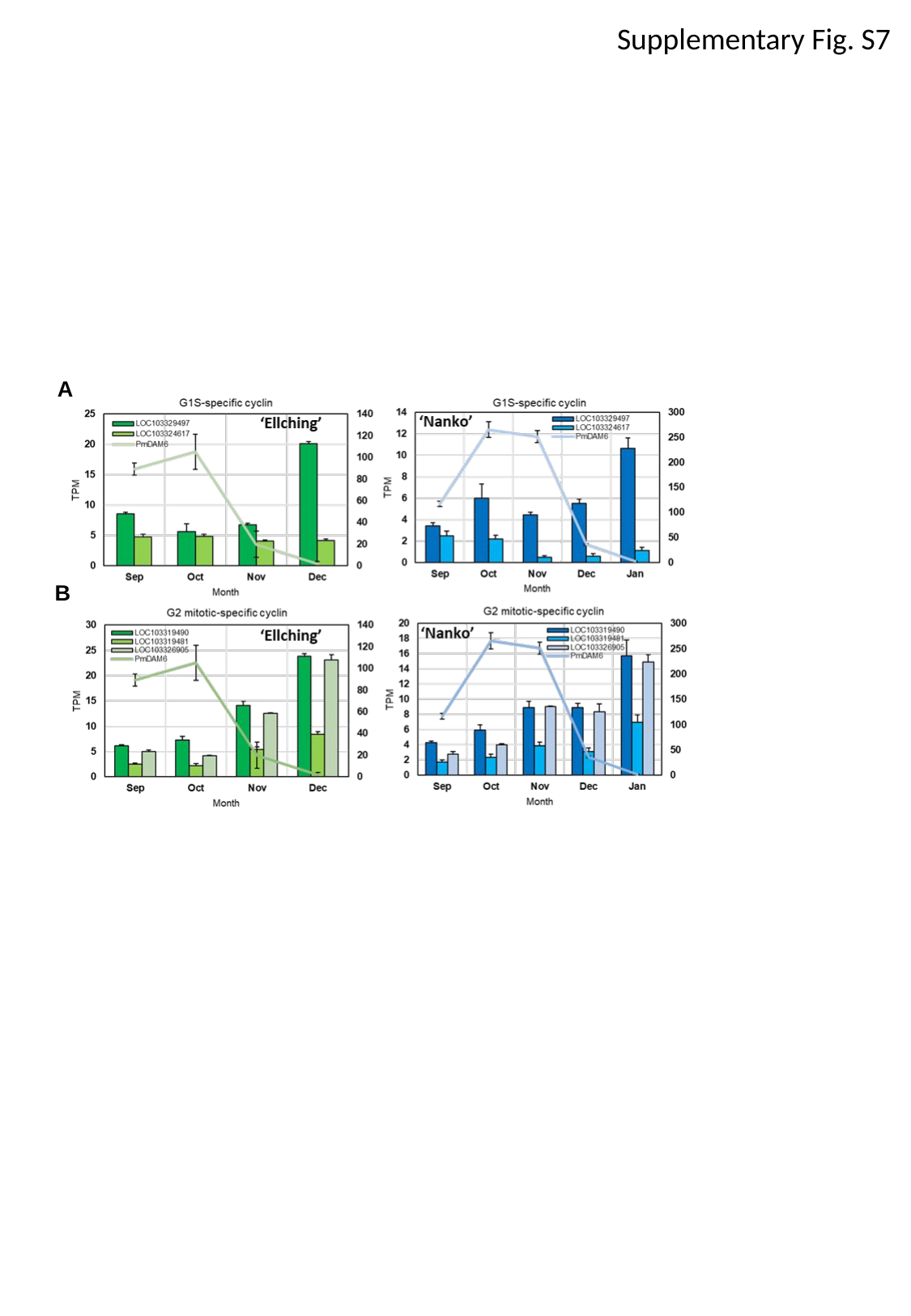

Supplementary Fig. S7
A
B

## Slide 14
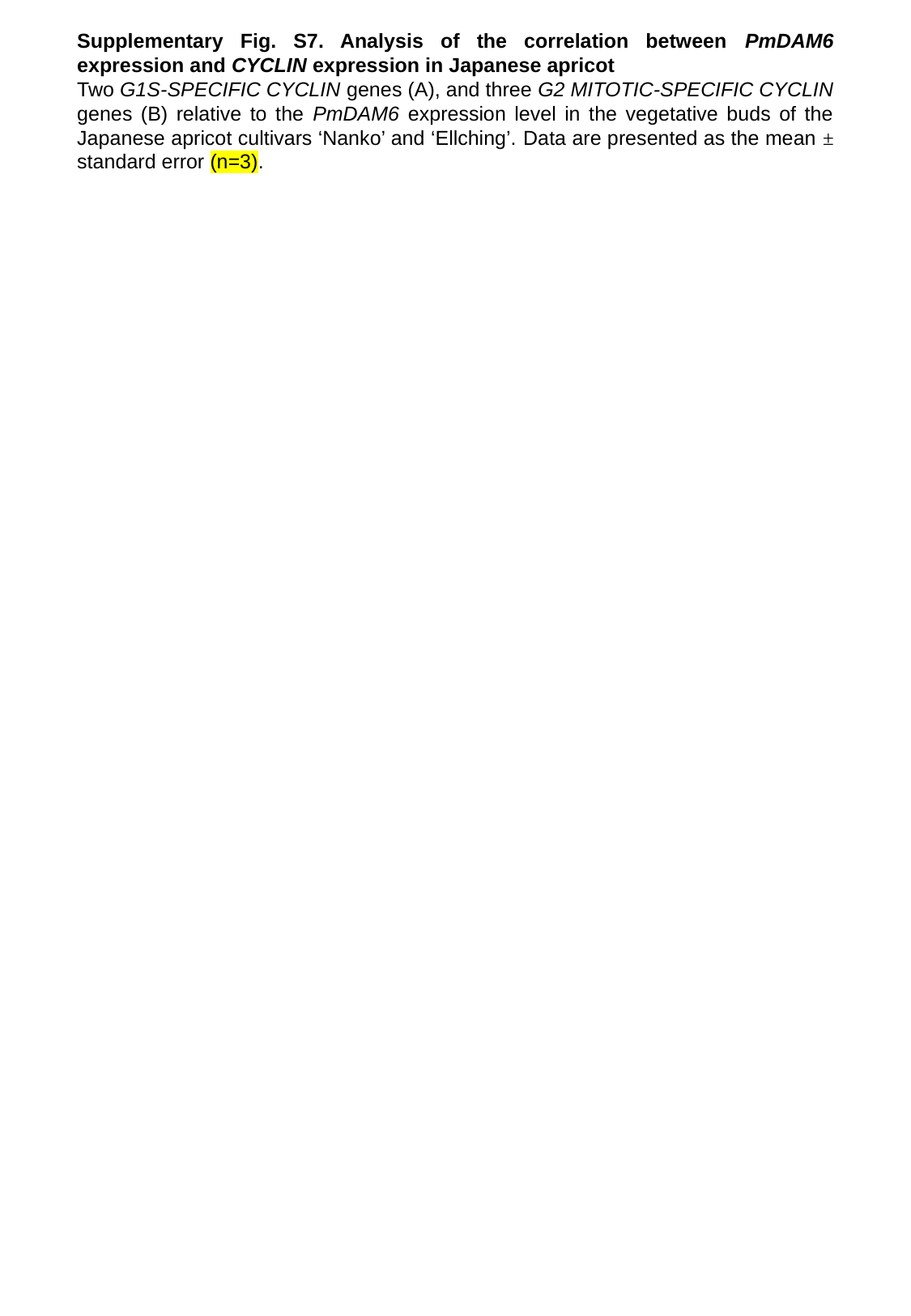

Supplementary Fig. S7. Analysis of the correlation between PmDAM6 expression and CYCLIN expression in Japanese apricot
Two G1S-SPECIFIC CYCLIN genes (A), and three G2 MITOTIC-SPECIFIC CYCLIN genes (B) relative to the PmDAM6 expression level in the vegetative buds of the Japanese apricot cultivars ‘Nanko’ and ‘Ellching’. Data are presented as the mean ± standard error (n=3).

## Slide 15
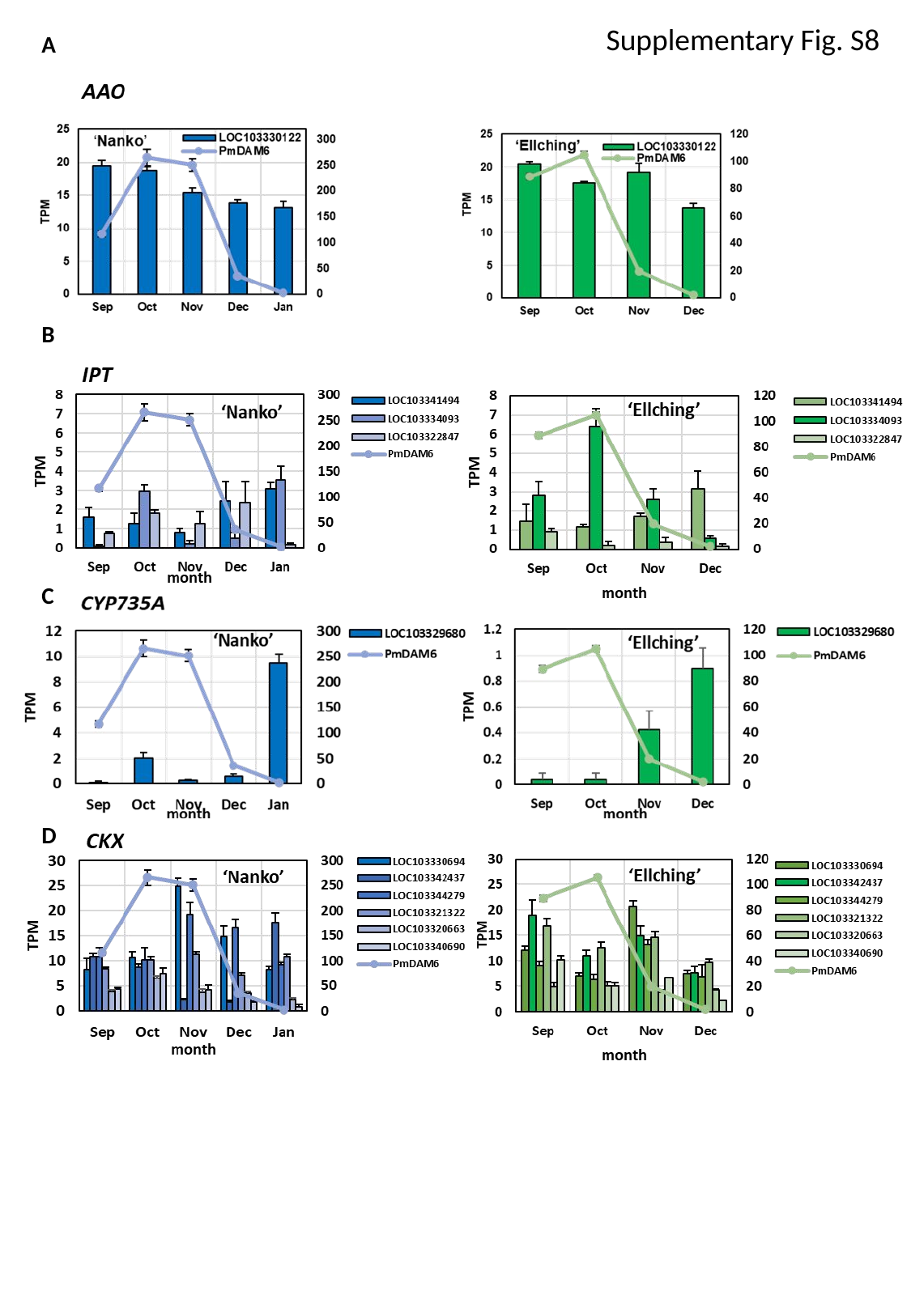

Supplementary Fig. S8
A
B
C
D

## Slide 16
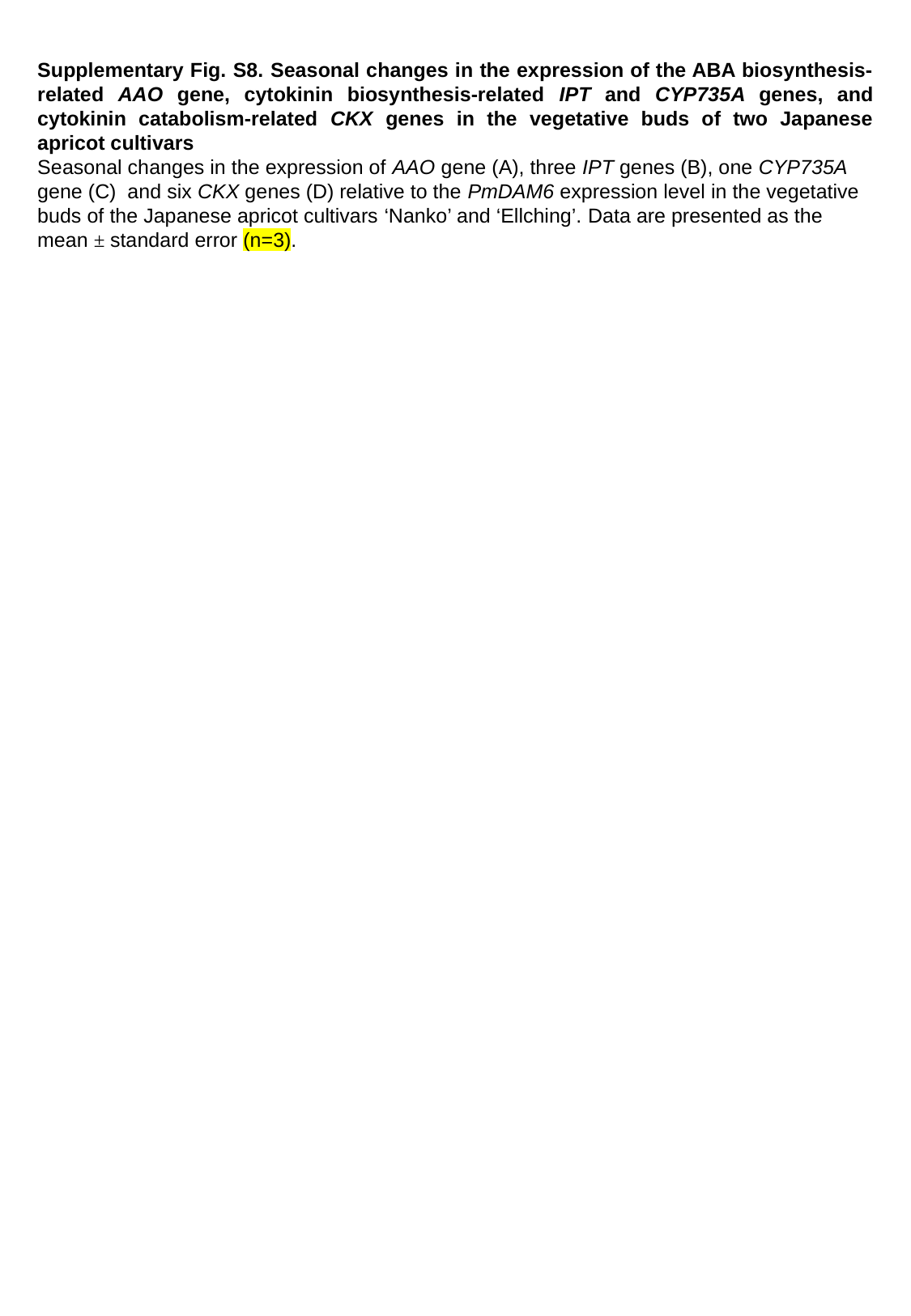

Supplementary Fig. S8. Seasonal changes in the expression of the ABA biosynthesis-related AAO gene, cytokinin biosynthesis-related IPT and CYP735A genes, and cytokinin catabolism-related CKX genes in the vegetative buds of two Japanese apricot cultivars
Seasonal changes in the expression of AAO gene (A), three IPT genes (B), one CYP735A gene (C) and six CKX genes (D) relative to the PmDAM6 expression level in the vegetative buds of the Japanese apricot cultivars ‘Nanko’ and ‘Ellching’. Data are presented as the mean ± standard error (n=3).

## Slide 17
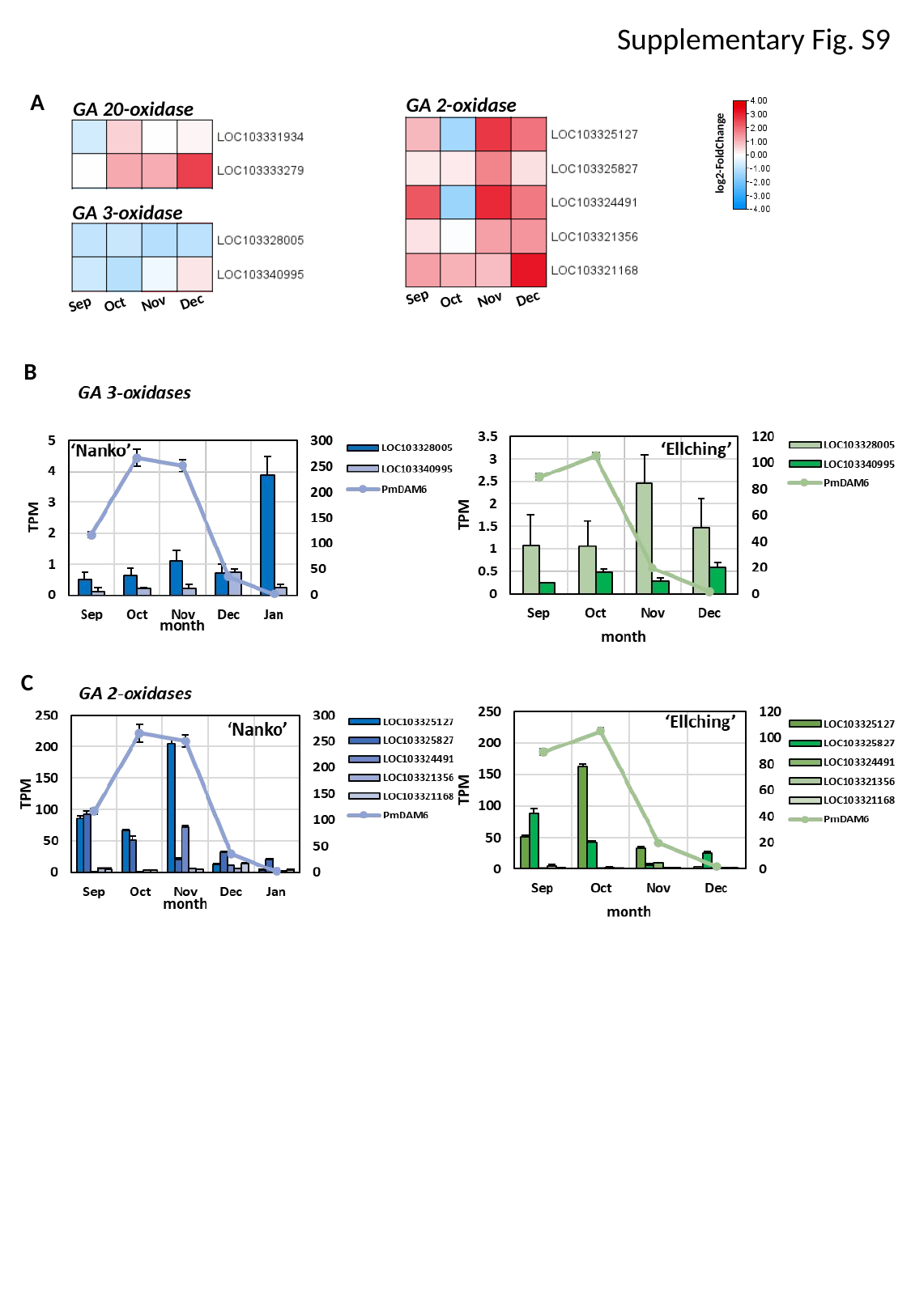

Supplementary Fig. S9
A
GA 2-oxidase
GA 20-oxidase
log2-FoldChange
GA 3-oxidase
Sep
Dec
Nov
Oct
Dec
Nov
Sep
Oct
B
C

## Slide 18
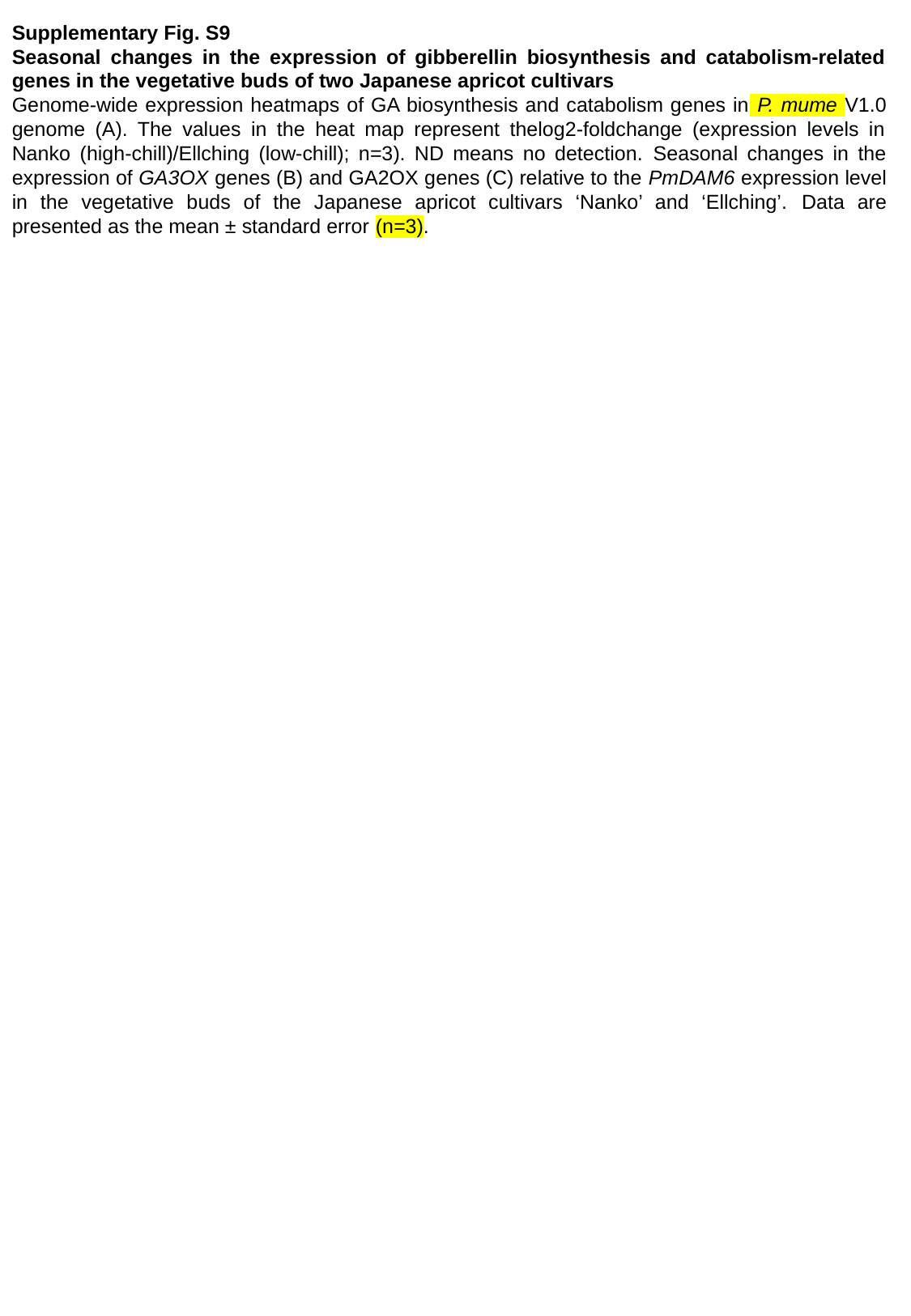

Supplementary Fig. S9
Seasonal changes in the expression of gibberellin biosynthesis and catabolism-related genes in the vegetative buds of two Japanese apricot cultivars
Genome-wide expression heatmaps of GA biosynthesis and catabolism genes in P. mume V1.0 genome (A). The values in the heat map represent thelog2-foldchange (expression levels in Nanko (high-chill)/Ellching (low-chill); n=3). ND means no detection. Seasonal changes in the expression of GA3OX genes (B) and GA2OX genes (C) relative to the PmDAM6 expression level in the vegetative buds of the Japanese apricot cultivars ‘Nanko’ and ‘Ellching’. Data are presented as the mean ± standard error (n=3).
